# Emergence of a floral colour polymorphism by pollinator-mediated overdominance

**DOI:** 10.1038/s41467-018-07936-x

**Published:** 2019-01-08

**Authors:** Roman T. Kellenberger, Kelsey J. R. P. Byers, Rita M. De Brito Francisco, Yannick M. Staedler, Amy M. LaFountain, Jürg Schönenberger, Florian P. Schiestl, Philipp M. Schlüter

**Affiliations:** 1 Department of Systematic and Evolutionary Botany, University of Zurich, Zollikerstrasse 107, 8008 Zurich, Switzerland; 2 Department of Plant and Microbial Biology, University of Zurich, Zollikerstrasse 107, 8008 Zurich, Switzerland; 3 Department of Botany and Biodiversity Research, University of Vienna, Rennweg 14, 1030 Vienna, Austria; 4 Department of Ecology and Evolutionary Biology, University of Connecticut, 75 North Eagleville Road, Storrs, CT 06269-3043 USA; 5 Institute of Botany, University of Hohenheim, Garbenstraße 30, 70599 Stuttgart, Germany; 6 Present Address: Department of Plant Sciences, University of Cambridge, Downing Street, Cambridge, CB2 3EA UK; 7 Present Address: Department of Zoology, University of Cambridge, Downing Street, Cambridge, CB2 3EJ UK

## Abstract

Maintenance of polymorphism by overdominance (heterozygote advantage) is a fundamental concept in evolutionary biology. In most examples known in nature, overdominance is a result of homozygotes suffering from deleterious effects. Here we show that overdominance maintains a non-deleterious polymorphism with black, red and white floral morphs in the Alpine orchid *Gymnadenia rhellicani*. Phenotypic, metabolomic and transcriptomic analyses reveal that the morphs differ solely in cyanidin pigments, which are linked to differential expression of an *anthocyanidin synthase* (*ANS*) gene. This expression difference is caused by a premature stop codon in an *ANS-*regulating *R2R3-MYB* transcription factor, which is heterozygous in the red colour morph. Furthermore, field observations show that bee and fly pollinators have opposite colour preferences; this results in higher fitness (seed set) of the heterozygous morph without deleterious effects in either homozygous morph. Together, these findings demonstrate that genuine overdominance exists in nature.

## Abstract (web summary)

Examples of overdominance are usually explained by deleterious effects in homozygotes. Here, Kellenberger et al. describe a case of overdominance in the floral color of the Alpine orchid G*ymnadenia rhellicani* apparently maintained by pollinator preferences without deleterious effects in homozygotes.

## Introduction

Overdominance is defined as a fitness advantage of individuals based on heterozygosity at a single locus^1^. Historically, overdominance was considered an important mechanism maintaining polymorphism in natural populations, as it provides a simple explanation for the retention of multiple alleles in a population^2,3^. Although a range of putatively overdominant cases have been identified, very few are documented well enough to (1) establish a clear connection between genotype, phenotype and the selective force, and (2) exclude any influence of other genetic loci or other selection pressures on the examined phenotypic trait^4,5^. The well-understood examples are usually associated with intense, short-term selective pressures such as host–pathogen interactions^6–8^ or artificial selection^4,9–15^. In these cases, homozygosity of the derived allele generally has a detrimental effect on fitness, and cessation of selection would revert the fitness advantage of the heterozygote. Overdominance is thus often seen as a rather short-lived phenomenon, and its perceived significance in the maintenance of polymorphism has somewhat diminished in favour of other modes of selection^16,17^.

Polymorphism of visual traits such as colour has been studied for over a century, as it is easily accessible and can provide insights into fundamental evolutionary processes^18^. In plants, polymorphism of floral display traits is often highly fitness relevant due to their impact on the interactions of a plant with pollinators^19,20^. Most cases of floral polymorphism have been associated with antagonistic pleiotropy^21–24^, negative frequency dependence^25,26^ or spatiotemporal heterogeneity^27–29^. To our knowledge, the action of heterozygote advantage has been proposed in the colour-polymorphic annuals *Cosmos bipinnatus*^30^, *Ipomoea* *purpurea*^31^ and *Sisyrinchium* sp.^32^ However, selection on the *Cosmos* colour morphs may not be strictly overdominant as the morphs also differ in number and size of flowers;^30^ likewise, pollinator-mediated selection in the other two species seems to be influenced by other factors (pleiotropic effects of the colour locus in *Ipomoea*^33,34^ and reproductive interference in *Sisyrinchium*^35^).

**Fig. 1:**
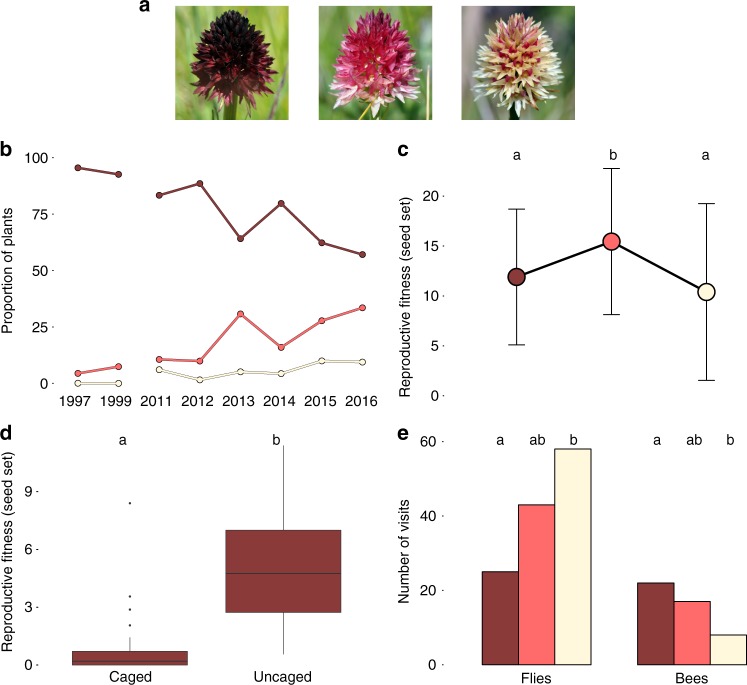
Ecological evidence for overdominance in *Gymnadenia rhellicani*. **a** Three *G. rhellicani* floral colour morphs are growing intermixedly at Puflatsch. **b** The fraction of red and white morphs is increasing at Puflatsch, suggesting a fitness advantage of the white, red or both morphs. **c** Red, intermediate plants have the highest reproductive fitness (ANOVA (*n* = 173), *F*(4, 168) = 8.935, *P* = 1.446 × 10^−6^ and Tukey's HSD post hoc test *t*_Black-Red_ = 3.334, *P*_Black-Red_ = 0.003, *t*_Black-White_ = −1.168, *P*_Black-White_ = 0.467, *t*_Red-White_ = −3.250, *P*_Red-White_ = 0.004); bars denote ± 1 standard deviation). **d** Shielding plants (ungrazed Ofenpass site) from pollinators yielded low seed set, indicating pollinator-dependent reproduction (two-sided *t* test (*n* = 42), *t*(34.818) = −4.939, *P* = 1.955 × 10^−5^, the outlier value was caused by an accidentally trapped grasshopper leaving a hole in the net); centre lines denote medians, bounds of boxes denote first and third quartiles, whiskers denote 1.5× interquartile ranges. **e** Time-lapse recordings (2017), showing bee preference for dark and fly preference for bright plants, maximising seed set in the red morph (*χ*^2^ test, Supplementary Fig. 1)

A yet unexplored floral colour polymorphism exists in a population of the Alpine orchid *Gymnadenia* (syn. *Nigritella*) *rhellicani* located on the Alpine plateau Puflatsch (Seiser Alm) in Northern Italy (Supplementary Table 1). Unlike in other locations, where the single inflorescences of *G. rhellicani* bear uniformly ‘black’ flowers, this population is strikingly polymorphic with 62% (wild type) ‘black’, 28% ‘red’ and 10% ‘white’ individuals (census 2015, Fig. 1a). Within the population, there is no difference in the spatial distribution between morphs, which often grow in close proximity (5 cm) to each other (Supplementary Fig. 1), and morphs do not switch colour throughout their perennial life cycle. Variation in floral coloration is thus likely genetically determined rather than a result of, for example, soil chemistry. To our knowledge, this population cannot be older than ca. 2000 years, when forests on Puflatsch were cleared;^36^ the first notion of the colour polymorphism is from 1906^37^, with a photographic record starting in 1971^38^. Moreover, frequency estimations conducted between 1997 and 2016 indicate an increase in relative abundance of the red and white colour morphs from total <5% to >40% (Fig. 1b, 1997–2014 data courtesy of Richard Lorenz). Although assessing evolutionary change from Hardy–Weinberg proportions in *Gymnadenia* populations is hampered by their pronounced age structure (cohorts of long-lived, periodically dormant individuals)^39^, the change in morph frequencies suggests the current action of strong selection pressure favouring the white, the intermediate or both alternate morphs in this system.

Here we use an eco-evo-devo approach to identify the genetic mechanism, the developmental basis and the evolutionary implications of the floral colour polymorphism in *G. rhellicani*. Taken together, our results suggest that this polymorphism has emerged by pollinator-mediated overdominance: the red colour morph is heterozygous for a single-nucleotide polymorphism (SNP), which introduces a premature stop codon in an *R2R3-MYB* transcription factor. This leads to reduced expression of an *anthocyanidin synthase* (*ANS*) gene, resulting in a reduction of red cyanidin pigment in flower tissue, apparently without affecting other phenotypic traits. The two main pollinator groups of this population, bees and flies, show opposite colour preferences and both visit the red colour morph in relatively high numbers. As a consequence, the red colour morph has the highest seed set and increases in abundance. Overdominance in *G. rhellicani* thus appears entirely based on colour, and unlike in most other overdominant systems in natural populations, the mutation does not seem to cause any deleterious effects in either of the homozygotes.

## Results

### Evolutionary implications of the colour polymorphism

To assess the selective forces maintaining the floral colour polymorphism in *G. rhellicani*, we first quantified both inflorescence survival (fraction of inflorescences eaten by insects and livestock as well as reappearance of inflorescences in the next season) and reproductive fitness (number of fruits × percentage of viable seeds) of 281 plants (Supplementary Table 2). While there was no difference in inflorescence survival between colour morphs (Supplementary Fig. 2a), red plants had a significantly higher seed set than either of the extreme colour morphs (Fig. 1c). This pattern could mainly be attributed to variation in the number of fruits (Supplementary Fig. 2b and c), which in orchids develop only after successful pollination^40^, supporting the hypothesis of pollinator-mediated heterozygote advantage. Flow cytometry of collected pollinia identified all colour morphs as diploid (Supplementary Fig. 2d), and shielding plants with glass fibre mesh showed that pollinators are indeed required for seed set (Fig. 1d). This rules out apomictic or autogamous reproduction as known from polyploid *Gymnadenia* species^41^.

*Gymnadenia rhellicani* is a nectar-producing plant visited by >60 different insect species from various orders^42,43^, and observations at Puflatsch have shown that pollination takes place during daytime with bees and flies as main carriers of pollinia (Supplementary Table 3). To test for differences in pollinator visitation, we (1) caught and identified insects on inflorescences in 2015, and placed ten cameras in front of (2) in situ pairs of black and red plants for four consecutive days in 2016, and (3) triplets of freshly cut inflorescences (one per colour morph) for 3 days in 2017, taking pictures every 60 s (2016: 25,007 frames; 2017: 14,419 frames). In 2016, we counted 160 landings of insects (18% bees, 81% flies and 1% other) and in 2017, we recorded 197 landings (24% bees, 64% flies and 12% other). While bees visit more dark than bright colour morphs, flies on the contrary visit more bright than dark colour morphs (Fig. 1e, Supplementary Fig. 2e, Supplementary Table 3). Although bees are less common, a limited survey in the population suggests that they are likely more efficient pollinators than flies (2 of 6 bees = 33% vs. 3 of 29 flies = 10% carried pollinia). Different preferences by bees and flies thus seem to exert opposite directional selection pressures on flower colour. The red, intermediate colour morph thereby attracts both pollinators in relatively high number, providing an explanation for the observed fitness advantage. Mapping of spectral reflectance data (*λ* = 300–700 nm) of flowers (Supplementary Fig. 2f) in the fly^44,45^ and bee^46^ visual spaces further suggested that both pollinator groups distinguish the colour morphs primarily based on luminance (Supplementary Fig. 2g and h).

### Phenotypic basis for pollinator choice

Considering these findings, bees and flies could either distinguish colour morphs by flower coloration intensity only or could rely on other phenotypic traits that correlate with colour. We therefore quantified a series of other potentially pollinator-relevant traits including plant height, flower number and inflorescence temperature in the morning and at noon, and also performed micro-computed tomography (µCT) of flowers to assess three-dimensional (3D) floral morphology. Our analyses showed that the three colour morphs do not differ in any of these traits (Supplementary Fig. 3a–d). We also collected and analysed floral volatiles by headspace sorption and gas chromatography mass-selective detection (GC-MSD) and assessed the composition of the floral volatile bouquet^47^. Neither the whole volatile bouquet nor the emission of individual scent compounds were different between the colour morphs (with the exception of one minor compound, Supplementary Figs. 3e and 4, Supplementary Table 4). A comparison of temporal volatile emission between 10:00, 15:30 and 21:00 further showed that all colour morphs reduce volatile emission after sunset (Supplementary Fig. 3f). *Gymnadenia rhellicani* thus does not use floral volatiles to attract crepuscular or nocturnal pollinators. Altogether, these results suggest that the underlying mutation(s) only affect(s) flower coloration rather than acting in a pleiotropic fashion on multiple traits. This further strengthens the argument that flower colour may be under overdominant selection.

### Developmental basis of the colour polymorphism

**Fig. 2:**
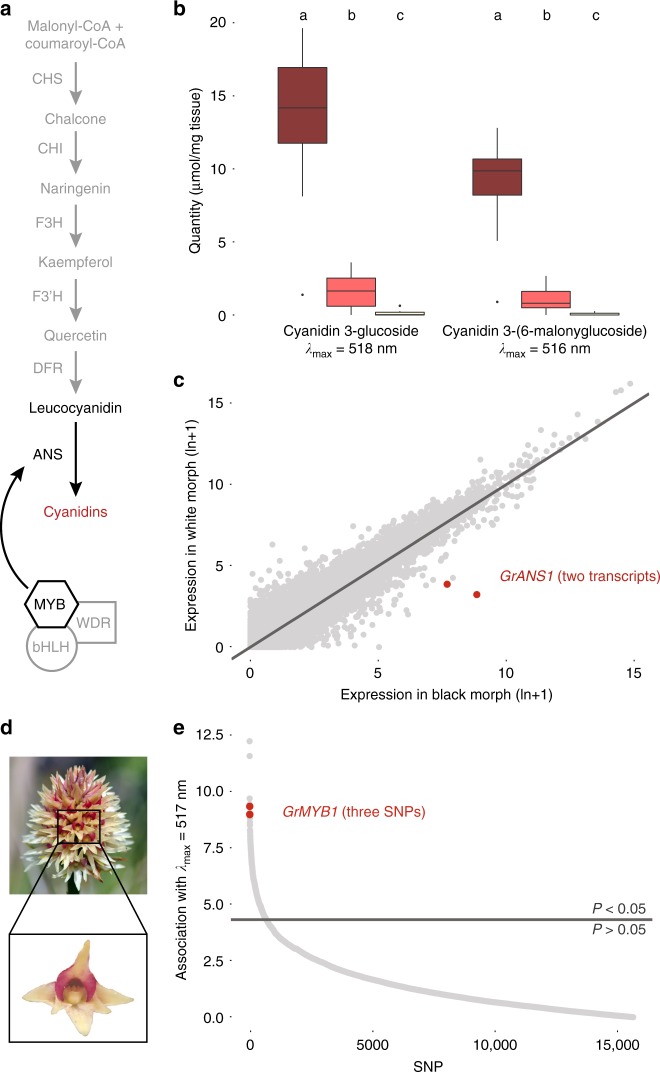
Molecular developmental basis of the colour polymorphism. **a** Simplified diagram of the cyanidin branch of the anthocyanin pathway (see Supplementary Fig. 5), showing ANS and its typical regulation by MYB-bHLH-WDR transcription factor complexes. **b** Concentrations of the two main anthocyanin pigments found in *G. rhellicani* flowers (ANOVA; see Supplementary Fig. 4, Supplementary Table 6); centre lines denote medians, bounds of boxes denote first and third quartiles, whiskers denote 1.5× interquartile ranges. **c** Two of the 13 transcripts significantly differentially expressed between black and white plants map to *GrANS1* (Supplementary Table 7). **d** Close-up of a ‘white’ flower; lateral labellum lobes still contain red pigment, suggesting a mutation in a regulator of spatial anthocyanin expression. **e** Transcriptome-wide association between SNPs and approximate cyanidin content (spectral reflectance): three top-10 SNPs occur in *GrMYB1* (Supplementary Table 8)

To characterise the molecular basis of this polymorphism, we combined the phenotypic measurements with metabolomic and transcriptomic data from the same individuals by extracting anthocyanin and carotenoid pigments as well as RNA from the same pool of four open flowers per individual. Quantification of anthocyanins and their colourless precursors with ultra-high-performance liquid chromatography (UHPLC-MS/MS) identified cyanidin-3-glucoside and a derivative, (putatively) cyanidin-3-(6-malonylglucoside), as dominant pigments in the flowers. Another derivative, peonidin-3-glucoside, was also present to a lesser degree. The abundance of the two main cyanidin compounds is more than 7.5-fold lower in red, and more than 30-fold lower in white plants than in the black morph (Fig. 2b, Supplementary Fig. 5, Supplementary Tables 5 and 6). In contrast, HPLC quantification of carotenoids showed that the flowers mainly contain β-carotene and lutein with no difference in concentration between the three morphs (Supplementary Fig. 6a). Next, we performed messenger RNA-sequencing (mRNA-seq) of one plant per colour morph, three other wild-type black *G. rhellicani* plants from other populations, and four individuals of three closely related *Gymnadenia* species (Supplementary Table 2). We de novo assembled the Illumina HiSeq 2500 reads into a combined reference transcriptome, yielding a total of 836,101 contigs with a N_50_ length of 553 bp. With an additional 45 plants from the focal Puflatsch population, 32 plants from other populations and 16 *Gymnadenia densiflora* plants we prepared multiplexed libraries for RNA 3′ expression profiling by low-coverage mRNA-seq (Illumina HiSeq 2500), resulting in 166 million reads that were then mapped to the reference transcriptome. We next performed differential expression analysis between all three morphs and found 13 transcripts with significantly different expression levels. Two of these, including the one with the highest statistical support, appeared to be derived from the same *anthocyanidin synthase* (*GrANS1*) gene. *ANS* encodes a key enzyme in the anthocyanin pathway (Fig. 2a, c, Supplementary Fig. 5, Supplementary Table 7).

### Genetic mechanism underlying the colour polymorphism

**Fig. 3:**
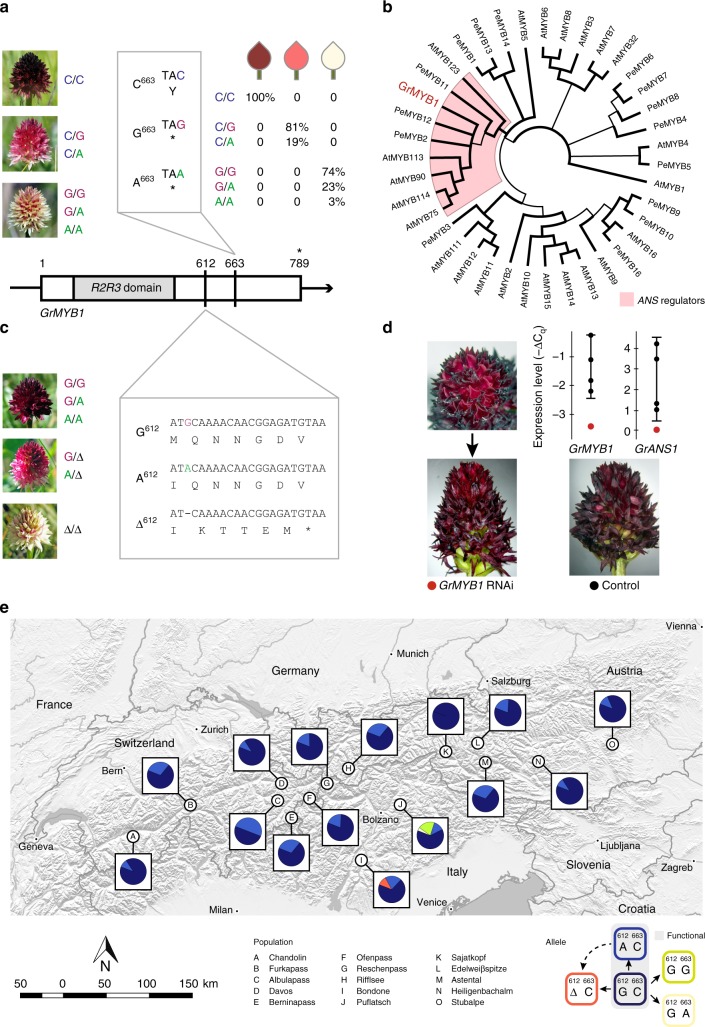
Genetic evidence for overdominance in *Gymnadenia rhellicani*. **a** Polymorphism at Puflatsch; red morph always heterozygous for functional variant (both alternative variants truncate the protein). **b** Phylogenetic analysis of *R2R3-MYB* domains (*Arabidopsis*, *AtMYB*; *Phalaenopsis*, *PeMYB*) places *GrMYB1* within *ANS* regulators (bold branches, posterior probability >80%). **c** Polymorphism at Bondone; derived variant (deletion/frameshift) is always heterozygous in red and homozygous in white morph. **d** RNAi of *GrMYB1*: partial pigment loss and reduced *GrMYB1* and *GrANS1* expression (red; controls black, 99% confidence intervals) in developing flowers. **e** Evolutionary relationships and relative frequencies of *GrMYB1* alleles (linked SNP positions 612 + 663) across the Alps; non-functional alleles at Puflatsch and Bondone. Map based on ALOS World 3D 30 m digital surface model from Japan Aerospace Exploration Agency, ©JAXA and administrative boundaries from GADM

A closer inspection of the white colour morph shows that the lateral lobes of the labellum still contain anthocyanin pigment (Fig. 2d), suggesting a mutation in an associated regulatory element rather than in the *GrANS1* gene itself. To find the underlying mutation, we identified SNPs from the expression profiling data and performed a transcriptome-wide association study (TWAS). Here, spectral reflectance at 517 nm wavelength was used as the phenotype, since this is the mean of the two main cyanidins’ absorption maxima (516 and 518 nm). While we did not find any associations in *GrANS1* itself (eight SNPs; *P* *=* 1 for all), three of the top ten SNP associations mapped to the 3′ non-coding region of the same *GrMYB1* gene, a member of the *R2R3-MYB* transcription factor family involved in the regulation of anthocyanin production (Fig. 2a, e, Supplementary Table 8). Since the expression profiles cover only the 3′-end of transcripts, these SNPs may not be causative themselves, but indicate the presence of alleles segregating according to colour morph. Closer inspection of the full *GrMYB1* transcript revealed a non-synonymous SNP with three allelic states: C (ancestral ‘wild-type’), G and A in the last exon of the coding region (Fig. 3a). Genotyping of all plants showed that black plants with high cyanidin content and *GrANS1* expression are always homozygous for the wild-type allele (C/C), white plants with low cyanidin content and *GrANS1* expression never contain the wild-type allele (G/G, G/A or A/A) and the red, intermediate colour morph is always heterozygous for the wild-type allele (C/G or C/A). Both transversions (C → G and C → A) introduce a premature stop codon, consistent with a loss of function of the derived alleles via truncating the protein by 43 amino acids. The observed segregation pattern is thus fully consistent with overdominance.

In several cases, follow-up studies on putative overdominant systems have shown that heterozygosity is not favoured due to a direct fitness effect of the identified locus, but rather due to its linkage to other loci under selection (associative overdominance)^48^. Although direct functional verification of an interaction of *GrMYB1* with the differentially expressed *GrANS1* gene is currently infeasible in *G. rhellicani*, we conducted follow-up investigations, which strongly indicate a direct effect of *GrMYB1* on plant colour patterning. First, we reconstructed the phylogenetic relationship of *GrMYB1* to other *MYB* gene copies from *Arabidopsis thaliana* and the orchid *Phalaenopsis*. Our analysis places *GrMYB1* within a clade consisting of *A. thaliana MYB* subgroup 6, and *Phalaenopsis equestris MYB 2*, *11* and *12*, all of which have been shown to regulate *ANS* expression patterning in these plants^49,50^ (Fig. 3b).

Second, we applied double-stranded RNA to cut inflorescences of wild-type (C/C) *G. rhellicani* plants from a natural population so as to reduce *GrMYB1* expression by RNA interference (RNAi). Of 22 treated plants (many of which were already at an advanced stage of flowering), one showed a weak and one a clear reduction in coloration of newly opening flowers, the expression of both *GrMYB1* and *GrANS1* being significantly decreased in the latter plant (Fig. 3d).

Third, we analysed the coding sequence of *GrANS1* in 30 individuals from Puflatsch and identified 4 putative alleles from a combination of 9 SNPs. *GrANS1* allelic state is neither associated with *GrANS1* expression levels nor plant colour, indicating *trans*-acting regulation of *GrANS1* (Supplementary Fig. 6c).

Fourth, we analysed the coding sequence of *GrMYB1* in 98 individuals from 14 other *G. rhellicani* populations distributed across the Alps (Supplementary Table 1). Most of these populations contain wild-type black plants with very rare occurrences of uniformly pink or yellow mutants (<0.1%), and both black and mutant plants contain only wild-type *GrMYB1* alleles (Fig. 3e). However, one smaller population located ca. 75 km south-west of Puflatsch at Monte Bondone, Italy (Supplementary Table 1), contains ca. 20% plants with a phenotype resembling the red and <1% plants with a phenotype resembling the white morph at Puflatsch (census 2015). Genotyping revealed that the nucleotide at an ancestral SNP position (R = G or A, encoding Met or Ile) in the last coding exon of *GrMYB1* is deleted (Δ) in alleles of all red and white plants analysed (Fig. 3c). Both the segregation pattern of this deletion (black: R/R; red: R/Δ; white: Δ/Δ) and its predicted effect on the protein (frameshift and truncation of 54 amino acids) are completely analogous to the Puflatsch population. Nevertheless, the two transversions discovered in the Puflatsch population do not occur at Bondone, and the deletion discovered at Bondone is absent at Puflatsch. This implies that *GrMYB1* must have mutated independently in the two populations, leading to parallel evolution of bright flowers in *G. rhellicani* (Fig. 3e).

## Discussion

Introduced into population genetics by T. Dobzhansky in 1951^51^, the concept of overdominance initially raised considerable controversy within the scientific community. Since then, interest in overdominance has waned because obtaining clear and complete evidence of overdominant selection in natural populations has been surprisingly difficult, especially at the genetic level^3^. In the few well-understood examples, heterozygotes usually have a fitness advantage because of deleterious effects in homozygotes, since (1) artificial breeding or pathogens select against wild-type homozygotes, and/or (2) the function of essential metabolic processes is impaired in derived homozygotes^17^. In contrast, overdominance appears to have evolved under different circumstances in *G. rhellicani*. The investigated colour polymorphism is not connected to breeding or diseases, and the mutation does not appear to have any detrimental effect in the homozygous state. Similar to the well-known example of heterozygote advantage at the major histocompatibility complex in vertebrates^52^, overdominance in *G. rhellicani* seems to be conditional on a single extrinsic factor (pollinator frequencies) instead of being maintained by superposition of an extrinsic with an opposing intrinsic factor.

Our study only lays the foundation for an understanding of the evolutionary and molecular processes in this system, and thus provides opportunities for further in-depth investigations: first, technical advances in ex situ propagation and genetic transformation of *G*. *rhellicani* may ultimately allow a direct functional verification of GrMYB1. Second, our study addresses plant–pollinator interactions from a plant’s perspective, but detailed pollinator behaviour and efficiency as well as the ultimate causes for colour preferences by the pollinators remain to be assessed. Third, our investigations were confined to flowering individuals over a few seasons, while long-term variation of selection as well as the effect of the mutation on other *G. rhellicani* life stages are currently unknown. However, pleiotropic effects are not expected to occur, as *GrMYB1* seems to be restricted in its expression pattern. Indeed, mutations in *R2R3-MYB* transcription factors are frequently involved in evolutionary transitions of flower coloration, probably because of their minimal pleiotropic effects^53^. This is also highlighted by the independent origin of the derived *GrMYB1* alleles in the two polymorphic *G. rhellicani* populations.

In conclusion, our study provides clear evidence of ordinary polymorphism maintained by overdominant selection. Overdominance therefore should not be discounted as an explanation for the frequent occurrence of polymorphism in natural populations.

## Methods

### Study sites and sampling strategies

The main polymorphic *G. rhellicani* population contains around 4500 individuals distributed mainly over two ridges running east–west on the volcanic outcrop of the Alm Puflatsch, Italy (Supplementary Table 1, detailed sample sizes are listed in Supplementary Table 2). The whole area is subject to intense browsing by cattle and horses during the summer, and lower parts of both ridges are usually mown in early autumn. To enable cross-comparisons, analyses included the same set of 48 plants distributed over both ridges whenever possible. The second polymorphic *G. rhellicani* population contains around 650 individuals and is situated on Monte Bondone, Italy (Supplementary Table 1). For the locations of all other wild-type populations see Supplementary Table 1. To keep track of individual plants in all populations over multiple seasons, they were invisibly marked by burying passive integrated transponder (PIT) tags (HPT23, Biomark, USA) in 1.5 ml centrifuge tubes close to the shoots. Re-localisation of tagged plants was done with a handheld Global Positioning System (GPS) receiver and a Biomark HPR Plus reader with a BP Plus portable antenna. All experiments were carried out with the required collection and CITES permits issued by the relevant authorities.

### Spatial distribution analysis

In July 2015, 14 plots were established in the Puflatsch population (Supplementary Fig. 1, Supplementary Table 1) with a dimension of 2 × 2 m, containing a total of 281 *G. rhellicani* plants (Supplementary Table 2). The plots were evenly distributed at 10 m intervals along the east–west axis of the entire Northern ridge, and a random azimuth of 0–5 m to the north or south. Within the plots, the position, height and colour of each flowering plant was recorded and all inflorescences were photographed. Spatial distribution of all *G. rhellicani* plants within all plots was modelled in *R* (*R* v.3.2.4, *R* Development Core Team 2016); randomness of the global spatial distribution pattern was assessed with a Kolmogorov–Smirnov Test, and differences in spatial distribution patterns between colour morphs were computed with a Studentised Permutation Test (999 random permutations)^54^, both implemented in the *R* package *spatstat* v.1.56-0^55^.

### Morph frequency estimations

Census data from 1997, 1999 and 2011–2014 (courtesy of Richard Lorenz) are based on counts of inflorescences along east–west transects through the entire population, while data from 2015 to 2016 are based on counts of inflorescences within the 14 plots established in the population (see above). To ensure cross-comparability of estimations obtained with both methods, morph frequencies were additionally estimated along east–west transects by K.J.R.P.B. in 2015 when the plots were set up.

### Fitness measurements

Number of flowers per inflorescence was inferred from the photographs of the plants within the 14 plots (see above) by hand-counting individual flowers with the aid of a custom marking script implemented in *R*. To correct for the fraction of flowers invisible on the photo, flower numbers were multiplied with a factor of 1.22, which was determined by comparing flower numbers between photos and actual inflorescences. In September 2015, the tagged plants were re-localised with a handheld GPS receiver (Garmin, Switzerland) and a Biomark HPR Plus reader with a BP Plus portable antenna. The number of eaten plants was recorded, differences in herbivory between morphs were computed in *R* with a *χ*^2^ test and the remaining infructescences were photographed. The number of capsules was inferred using the aforementioned custom *R* script. To assess seed quality, the content of five capsules per individual were photographed under a microscope with transmitted light and ×20 magnification (Carl Zeiss, Germany). Since orchid seeds have a transparent seed coat and do not contain an endosperm, seed viability can be estimated by assessing the presence of an embryo. Viability was estimated from 100 seeds per plant using the marking script. Number of capsules was multiplied by relative seed viability to produce reproductive fitness values. Resulting values >0 were BoxCox transformed using the *R* package *caret* v.6.0-76. Relative fitness differences between morphs were then computed in *R* applying a Tukey's honest significance (HSD) post hoc test on an Akaike information criterion optimised linear model with colour, standardised plant height and standardised flower number as fixed effects. In July 2016, the plots were revisited and differences in re-flowering between morphs were computed in *R* with a *χ*^2^ test.

### Pollinator exclusion

To avoid incidents with livestock at Puflatsch, this experiment was conducted in an ungrazed wild-type population on top of Ofenpass, Switzerland (Supplementary Table 1). The SNP data derived from low-coverage mRNA-seq (see below) suggests that this population shares its genetic ancestry with the colour-polymorphic population at Puflatsch (not shown). In June 2016, 40 plants in bud stage were randomly selected and PIT tagged, and 20 plants were covered with white glass fibre nets (1 mm mesh size) attached to cylindrical metal frames of 25 cm height × 10 cm diameter. In September 2016, capsules were counted and seed quality was determined as described above. Fitness differences were computed in *R* with a two-sided *t* test.

### Pollinator recordings

On the 11th of July 2015, insects observed on *G. rhellicani* inflorescences along east–west transects on both ridges were caught with nets in the morning (10:00) and after sunset (22:00), identified and checked for pollinia. During peak flowering in July 2016, ten Somikon (Pearl, Switzerland) and one Brinno TLC 200 (Brinno, Taiwan) time-lapse camera were placed in front of in situ pairs of black and red plants growing within ca. 10 cm distance of each other at the Puflatsch site. The plants were recorded at 1 min intervals during four consecutive days, with cameras shutting off automatically at night. Two people (R.T.K. and K.J.R.P.B.) independently screened all images for number and type of pollinators. In July 2017, pollinator recordings were repeated for three consecutive days: three freshly cut inflorescences (one per morph, randomly chosen) were randomly placed in ca. 5 cm distance of each other in buried 15 ml tubes filled with water in front of each camera (9 Somikon and 1 Brinno). Differences in visitation of pollinator groups between morphs were calculated in *R* with Holm-adjusted *χ*^2^ tests.

### Flow cytometry

Ploidy analysis was based on a protocol in Gross and Schiestl^56^ with minor modifications. Pollinia from three flowers per individual were ground in 200 μl Otto 1 buffer (0.1 M citric acid monohydrate in 0.5% Triton X-100) in a 1.5 ml reaction tube using a pestle. An internal standard solution was prepared by grinding 2 cm^2^ leaf tissue of *Phaseolus coccineus* ‘Scarlett Emperor’ (Sativa Rheinau AG, Switzerland) in 1 ml Otto 1 solution, and filtering the suspension through a 30 μm CellTrics filter (Sysmex, Germany). After addition of 5 μl of this standard solution, each sample was filtered through a 30 μm CellTrics filter and centrifuged for 5 min at 380 × *g*. The supernatant was removed, and nuclei were resuspended in 40 μl Otto 1 buffer. Samples were analysed on a Beckman Coulter Cell Lab Quanta flow cytometer (Beckman Coulter, USA) loaded with 160 μl Otto 2 solution (0.4 M disodium phosphate heptahydrate with 4 μg ml^−1^ 4′,6-diamidine-2′-phenylindole dihydrochloride added before usage). Ploidy was determined as the ratio between the medians of the standard and sample peaks in the flow cytometric histogram. As *G. rhellicani* is generally diploid^41^, the lowest sample peak detected was assumed to represent the haploid pollinaria of diploid individuals, and the two adjacent peaks correspond to the diploid and (if present) tetraploid state.

### Spectral mapping

Spectral measurements were taken with an AvaSpec spectrometer (Avantes, Netherlands) from three flowers per individual. The three spectral curves per sample were averaged between 300 and 700 nm wavelength, smoothed and normalised. The curves were mapped into the fly visual space using a custom *R* script based on Troje^44^, and into the bee visual space using an *R* script published by Sedeek et al.^57^ with minor modifications.

### Temperature measurements

Inflorescence temperature was measured in the morning between 09:00 and 10:00 (60 plants) and in the afternoon between 12:00 and 13:00 (90 plants) on a cloudless day in July 2016. Temperatures were recorded with an infrared thermometer (Model JHK-6606, Walter Werkzeuge, Austria) in two transects from east to west along the bottom and top part of the Northern ridge.

### Micro-computed tomography

In July 2014, one flower from the lower part of the inflorescence was collected and stored in 1.5 ml FAA fixative (50% ethanol, 5% glacial acetic acid, 3.7% formaldehyde). Flowers were infiltrated twice with a contrast solution (1% phosphotungstic acid in FAA) and subsequently embedded in acryl pillow foam (DACRON^®^COMFOREL^®^, Invista, USA) according to Staedler et al.^58^. 3D flower morphology was obtained by µCT, using a MicroXCT-200 imaging system (Xradia, USA) with a 90 kV Microfocus X-ray. All samples were scanned with the following parameters: acceleration voltage, 37 kV; source current, 200 µA; exposure time, 1.5 s; number of exposures, 728; camera binning, 4; objective, large field of view (magnification = ×0.4); voxel size, 38.7475 µm. 3D data were exported in DICOM format. In *Amira* v.5.3.3 (Zuse Institute, Germany), a total of 21 geometric landmarks were positioned on each scanned flower: one at the base and tip of each petal, labellum and column, three along the left and right side of the labellum, one in the centre of the labellum and one at the tip of the spur (Supplementary Fig. 3d). Landmark positions were then compared among the three colour morphs with Procrustes ANOVA (analysis of variance) from the *R* package *geomorph* v.3.0.3^59^.

### Floral volatile analysis

Volatile organic compounds (VOC) were collected with non-destructive headspace sorption, a standard method also used in other *Gymnadenia* studies^60^. Entire inflorescences were enclosed in Toppits PET oven bags (Cofresco Frischhalteprodukte, Germany) and attached to a battery-driven pump connected to a glass tube loaded with 20 mg Tenax TA (60/80 mesh, Supelco, USA). Air was pulled from the bag through the Tenax filter at a flow rate of 100 ml min^−1^ for 40 min. Background VOC levels were determined with simultaneous samples from empty oven bags. Analysis of VOC samples was done using GC-MSD as described in Gervasi and Schiestl^61^. Identification and quantification of compounds was performed with the MSD ChemStation program E. 02.02 (Agilent, USA) based on a mass spectral library built on calibration curves using three to five different concentrations of authentic reference standards. To assess possible temporal fluctuations in intensity and composition of the scent bouquet, VOCs of 14 additional plants on the Southern ridge were collected at three different time points (10:00, 15:30 and 21:00) in July 2015 and analysed as described above. Emission of the 15 compounds contributing >0.1% to the floral bouquet was converted to pgl^−1^ air and BoxCox transformed to approach normality using the *R* package *caret* v.6.0-73^62^. Differences in VOC emission between colour morphs were compared with (1) principal component analysis, and (2) Holm-adjusted linear mixed-effects models with sampling year as random factors and Tukey's HSD post hoc tests (*R* packages *nlme* v.3.1-131^63^ and *multcomp* v.1.4-6^64^).

### UHPLC-MS/MS of anthocyanins

In July 2014, four flowers per plant were collected from the top, centre and bottom part of the inflorescence, respectively. Flowers were flash frozen in liquid nitrogen and ground to powder using a tissue lyser (Retsch Technology, Germany). Fifty percent of each sample were weighed, transferred to a 2 ml tube and flavonoids were extracted in 500 μl of 80% methanol (MeOH). Samples were ground again in solution, incubated overnight at 4 °C and centrifuged at 11,000 × *g* and 4 °C for 10 min. Supernatants were transferred to 1.5 ml tubes, evaporated in a Savant SpeedVac concentrator (Thermo Fisher Scientific, USA) at 42 °C and resuspended in 100 μl 50% MeOH + 0.1% formic acid. After sonication for 5 min, samples were centrifuged at 11,000 × *g* and 4 °C for 5 min, and transferred to liquid chromatography vials. Samples were run on a UHPLC (Dionex UltiMate 3000, Thermo Fisher Scientific, USA) coupled to a Bruker compact electrospray ionisation-quadrupole-time-of-flight tandem-mass spectrometer (Bruker Daltonics, USA). The UHPLC separation was performed with a C18 reverse-phase column (ACQUITY UPLC TM BEH C18, 1.7 µm, 2.1 × 150 mm, Waters, USA) at 28 °C using the following gradient of solvent B (acetonitrile with 0.1% (v/v) formic acid) and solvent A (water with 0.1% (v/v) formic acid): 0–0.5 min, 5% B; 0.5–12 min, 5–100% B; 12–14 min, 100% B; 14–16 min, 100–5% B. The flow rate was set up to 0.3 ml min^−1^ and 5 µl of each sample was injected. The ESI source was operated in positive mode and parameters were set as follows: gas temperature, 220 °C; drying gas, 9 l min^−1^; nebuliser, 2.2 bar; capillary voltage, 4500 V; end plate offset, 500 V. The instrument was set to acquire an *m*/*z* range of 50–1300. Conditions for MS/MS were set as described by Christ et al^65^. All data were recalibrated internally using pre-run injection of 10 mM sodium hydroxide in 0.2% formic acid, 49.8% water, 50% isopropanol (v/v/v). Data Analysis v.4.2 (Bruker Daltonics, USA) and TargetAnalysis v.1.3 (Bruker Daltonics, USA) were used to analyse the data. Absolute flavonoid quantification was based on standard curves and this analysis was performed using QuantAnalysis v.2.2 (Bruker Daltonics, USA). Flavonoids were identified and annotated by comparison with authentic reference standards data, specifically: *m*/*z* and MS/MS data, ultraviolet spectrum and retention time profiling except for cyanidin-3-(6-malonylglucoside), for which no reference standard was available. This compound was tentatively identified by an interpretation of the data spectrum obtained and based on previous results^66^. The identified peaks were integrated and standardised by flower tissue weight and the molecular weight of the compound. Concentrations of the two main compounds cyanidin-3-glucoside and cyanidin-3-(6-malonylglucoside) were calculated based on a calibration curve of cyanidin-3-glucoside. For comparison between morphs, standardised quantities were BoxCox transformed and assessed compound-wise with Holm-adjusted ANOVA and Tukey's HSD post hoc tests using the *R* package *caret* v.6.0-76^62^.

### HPLC of carotenoids

Carotenoids were re-extracted from the anthocyanin samples (see above). Eighty microlitres of hexane were added to each sample and the solution was vortexed and incubated on ice for 10 min. After centrifugation for 2 min at 11,000 × *g*, the upper phase was removed with a glass syringe and transferred to an amber glass vial (Supelco, USA). Samples were evaporated in a Savant SpeedVac concentrator (Thermo Fisher Scientific, USA) and resuspended in 100 μl diethyl ether. As described by LaFountain et al.^67^, the samples were subjected to saponification prior to analysis: 100 μl of 5% ethanolic KOH was added, and the samples were vortexed and incubated for 2 h in the dark at 21 °C. After addition of 400 μl hexane:diethyl ether (1:1), samples were washed with 500 μl H_2_O and placed on ice for phase separation. Washing of samples was repeated ca. 4–5 times until the pH of the aqueous phase appeared neutral on pH indicator paper. The upper phase was transferred to a vitreous UHPLC vial (Infochroma, Switzerland) and evaporated in a SpeedVac concentrator. Samples were re-solubilised in 60 μl acetonitrile:MeOH:H_2_O (87:10:3) and analysed on a C18 Hypersil ODS column (250 × 4.6 mm; Thermo Fisher Scientific, USA) at 25 °C using the following gradient of solvent A (20% 1 M ammonium acetate, 80% MeOH) and B (20% acetone, 80% MeOH): 0–15 min, 0–100% B; 15–25 min, 100% B; 25–28 min, 100–0% B; 28–32 min, 0% B. The flow rate was set up to 1 ml min^−1^ and 40 µl of each sample was injected. Parameters were controlled by a Gynkotek liquid chromatography system (Thermo Fisher Scientific, USA) equipped with a UVD340S diode array detector set at 450 nm for carotenoid identification. The two main carotenoids β-carotene and lutein were identified and quantified as described for the anthocyanins using calibration curves of authentic reference standards.

### Transcriptome assembly

mRNA-seq was performed for one individual per morph from Puflatsch, one individual from Ofenpass, Switzerland, two individuals from Chandolin, Switzerland, one *G. densiflora* plant from each of two populations near Tschierv, Switzerland and Davos Dorf, Switzerland, one *G. odoratissima* plant from a population in the Münstertal, Switzerland and one *G. conopsea* plant from a population near Cinuos-chel-Brail, Switzerland (the latter two courtesy Karin Gross). RNA was extracted from the remaining 50% of the ground flowers used for UHPLC-MS/MS (see above). Total RNA was extracted with TRIzol reagent (Thermo Fisher Scientific, Massachusetts) according to the manufacturer’s protocol and subsequently purified using a Qiagen RNeasy MinElute Cleanup Kit (Qiagen, Netherlands). RNA was quantified on a 2100 Bioanalyzer (Agilent Technologies, USA) and paired-end sequenced on one lane of an Illumina HiSeq 2500 sequencer (Illumina, USA). mRNA-seq reads were trimmed using *Trimmomatic* v.0.36^68^ to remove Illumina adapters, leading and trailing bases below quality 3, to truncate reads when average quality in a 4-bp sliding window dropped below 15 and finally to discard reads below 36 bp in length. Surviving reads were then de novo assembled to transcripts using *Trinity* v.2.0.6^69^.

### Generation of mRNA expression profiles

Expression profiling was conducted for 45 plants from Puflatsch, for 26 plants from Chandolin and for 6 plants from Ofenpass (see Supplementary Table 1 for population locations). Total RNA was extracted from the other half of the ground flower tissue used for UHPLC-MS/MS (see above) using TRIzol according to the manufacturer’s protocol. Samples were quantified with a Qubit fluorometer and RNA HS Assay Kit (Thermo Fisher Scientific, USA), and libraries were prepared with a Lexogen QuantSeq 3′ mRNA-Seq Kit (Lexogen, Austria) according to the manufacturer’s protocol. All libraries were pooled and sequenced single-end on one lane of an Illumina HiSeq 2500 sequencer.

### Differential expression analysis

Raw expression profile reads were trimmed to a minimal length of 25 bp with *Trimmomatic* v.0.36^68^. Trimmed reads were then mapped to the reference transcriptome, and expression levels were estimated using *RSEM* v.1.2.31^70^ and *bowtie2* v.2.2.9^71^ as aligner. Posterior mean counts per morph were subsequently extracted with a custom *Perl* script. Transcripts with fewer than 0.5 counts per million reads in at least 10 samples were removed, and negative binomial generalised log-linear models were fitted for transcripts with at least a two-fold expression difference using *edgeR* v.3.12.1^72^. Transcripts with a significant expression difference were searched against the NCBI nucleotide database (nr) with *blastn*^73^.

### Transcriptome-wide association study

Trimmed expression profile reads (see above) were mapped to the indexed reference transcriptome with *bowtie2* v.2.2.9^71^ and converted to indexed bam files. Variant calling of these files was performed with *samtools* v.1.3^74^ excluding bases with a quality of <13. The output was indexed and converted to vcf format using *bcftools* v.1.3^74^. Subsequently, conversion from vcf to hapmap format was performed in *R* v.3.2.4 using the *biOP4R* library function bdVcf2Hapmap (https://sourceforge.net/projects/biop) under exclusion of variants with a minimal depth of <15. Normalised spectral reflectance at 517 nm wavelength was extracted for each sample from the measured spectral curves (see above), and transcriptome-wide association of variants with the spectral data was assessed by fitting compressed mixed linear models with *GAPIT* v.2^75^, including the first seven principal components. Sequences of the ten transcripts with the strongest association were searched against the NCBI nucleotide database (nr) with *blastn*^73^.

### Phylogenetic analysis

Coding sequences of *Arabidopsis thaliana MYB1-16*, *32*, *75*, *90*, *111*, *113*, *114* and *123* and *Phalaenopsis equestris MYB1-14* and *MYB1-16* were retrieved from GenBank and aligned at protein level with *GrMYB1* using *MEGA* v.7^76^. The sequence of the conserved *R2R3* domain was extracted and used for phylogenetic analysis with *MrBayes* v.3.2.6 as described by Streisfeld and Rausher^77^. In brief, the run was performed with default settings for priors until apparent stationary of log likelihood was reached (two million generations with a sample frequency of 500, and a diagnosis frequency of 5000).

### *GrMYB1* genotyping

Genotyping of *GrMYB1* was conducted for three sites in Italy: 88 plants from Puflatsch, 23 plants from Monte Bondone, 16 plants from Reschenpass; five sites in Switzerland: five plants each of populations from Chandolin, Furkapass, Berninapass, Davos and Ofenpass; and four sites in Austria: Rifflsee, Astental and Heiligenbachalm, and four plants from Stubalpe (Supplementary Tables 1 and 2). Total RNA was extracted with TRIzol reagent as described above, and 1 µg of RNA was treated with DNAse I (Thermo Fisher Scientific, USA) according to the manufacturer’s protocol, and then converted to complementary DNA (cDNA) using Revert-aid H^−^ Reverse Transcriptase (Thermo Fisher Scientific, USA) according to the manufacturer’s protocol. The *GrMYB1* coding sequence was amplified with Phusion HotStart II polymerase (Thermo Fisher Scientific, USA); PCR: 4 µl HF buffer (5×), 0.4 µl dNTPs (10 mM each), 2 µl *GrMYB1*-F/F2 primer (5 µM, Supplementary Table 9), 2 µl *GrMYB1*-R primer (5 µM, Supplementary Table 9), 0.2 µl polymerase, 1 µl cDNA, 10.4 µl ddH_2_O; PCR conditions: 98 °C for 30 s, 35 cycles of 98 °C for 10 s, 62.6 °C for 30 s, 72 °C for 30 s, followed by 72 °C for 5 min. For cleanup, 5 µl of PCR reaction were mixed with 0.5 µl of Exo I (20U µl^−1^) and 2 µl FastAP (1U µl^−1^, both Thermo Fisher Scientific, Massachusetts), and incubated at 37 °C for 20 min followed by inactivation at 85 °C for 15 min. Clean PCR reactions were Sanger sequenced on an Applied Biosystems 3130xl genetic analyser using BigDye™ Terminator v3.1 Cycle Sequencing chemistry (Thermo Fisher Scientific, USA). Sequencing PCR (10 µl) used 2 µl sequencing buffer (5×), 1 µl *GrMYB1*-F/F2/R primer (5 µM, Supplementary Table 9), 1 µl BigDye™ v.3.1, 1 µl clean PCR reaction, 5 µl ddH_2_O and the following PCR conditions: 96 °C for 2 min, 30 cycles of 96 °C for 10 s, 50 °C for 5 s, 60 °C for 4 min. For plants with no or degraded RNA available, DNA was extracted from leaf tissue dried in silica gel using a Qiagen DNEasy Plant Mini Kit (Qiagen, Netherlands) according to the manufacturer’s protocol, and a 385 bp fragment in the last coding exon was amplified and sequenced as described above. Relative peak heights (fluorescence intensities) of each potential nucleotide (A, T, C, G) at the SNP positions were extracted from the.ab1 trace files using a custom *R* script based upon the *biOP4R* library (https://sourceforge.net/projects/biop) functions bdAbifReadFile, bdAbifGetPeakLoc and bdAbifGetTrace. Nucleotides with a relative fluorescence intensity of ≥20% were counted as present, and the genotype of a plant was recorded as the combination of nucleotides present.

### *GrANS1* genotyping

Genotyping of *GrANS1* was conducted for 30 plants from Puflatsch with data on *GrMYB1* genotype, *GrANS1* expression and sufficient cDNA left for PCR amplification. The *GrANS1* coding sequence was amplified in two parts (517 and 599 bp) using internal primers; PCR set-up: 4 µl HF buffer (5×), 0.4 µl dNTPs (10 mM each), 2 µl *GrANS1*-F/Fi primer (5 µM, Supplementary Table 9), 2 µl *GrANS1*-Ri/R primer (5 µM, Supplementary Table 9), 0.2 µl polymerase, 1 µl cDNA, 10.4 µl ddH_2_O; PCR conditions: 98 °C for 30 s, 35 cycles of 98 °C for 10 s, T_A_[F-Ri] = 65.3 °C/T_A_[Fi-R] = 66.1 °C for 30 s, 72 °C for 30 s, followed by 72 °C for 5 min. PCR reactions were diluted 1:4 in ddH_2_O and Sanger sequenced using primers *GrANS1*-Ri/Fi at the DNA Sequencing Facility, Department of Biochemistry, University of Cambridge. Nine SNP positions were identified from these sequences and the *G. rhellicani* reference transcriptome (positions 67, 258, 593, 810, 998, 1008, 1023, 1029 and 1073 in the coding sequence). Genotyping was conducted as described above.

### Production of *GrMYB1* dsRNA

The coding sequence of the last exon of the wild-type *GrMYB1* allele was amplified with Phusion HotStart II polymerase (Thermo Fisher Scientific, USA); PCR: 4 µl HF buffer (5×), 0.4 µl dNTPs (10 mM each), 2 µl *GrMYB1*-attB-F primer (5 µM, Supplementary Table 9), 2 µl *GrMYB1*-attB-R primer (5 µM, Supplementary Table 9), 0.2 µl polymerase, 1 µl cDNA, 10.4 µl ddH_2_O; PCR conditions: 98 °C for 30 s, 5 cycles of 98 °C for 10 s, 65 °C for 30 s, 72 °C for 40 s, 35 cycles of 98 °C for 10 s, 72 °C for 40 s, followed by 72 °C for 5 min. PCR reactions were cleaned up with a NucleoSpin^®^ Extract II Kit (Macherey-Nagel, Germany) according to the manufacturer’s protocol. The amplicons were cloned into a pDONR207 vector via GATEWAY™ cloning (Thermo Fisher Scientific, USA): 75 ng of cleaned PCR fragment was combined with 75 ng pDONR207 and 1 µl BP clonase II, and ddH_2_O was added to a total of 5 µl. Reactions were incubated at 25 °C for 2 h, 0.5 µl proteinase K (2 µg µl^−1^) was added, and reactions were incubated at 37 °C for 10 min. Chemo-competent *Escherichia coli* DH5α (Thermo Fisher Scientific, USA) were transformed with this construct using a standard protocol, and plasmids were extracted with a QIAprep^®^ Spin Miniprep Kit (Qiagen, Netherlands) according to the manufacturer’s protocol. In a second step, the *GrMYB1* fragment was cloned into a pL4440 vector, a gift of Andrew Fire (plasmid #1654, Addgene, USA). To do so, 50 ng of pDONR207 construct were combined with 75 ng pL4440, 1 µl LR clonase II and ddH_2_O was added to 5 µl. The reaction was incubated as described above, and chemo-competent *E. coli* HT115 (courtesy of Anita Dirks) were transformed with this construct using a standard protocol. Double-strand RNA (dsRNA) was produced according to Lau et al.^78^ with some modifications: after overnight incubation at 37 °C/220 rpm in 5 ml lysogeny broth medium with 50 µg ml^−1^ ampicillin and 10 µg ml^−1^ tetracycline, the culture was diluted 1:100 in 500 ml 2× yeast tryptone medium with the same concentration of antibiotics, and incubated at 37 °C/220 rpm. At OD_600_ = 0.7, 2 ml of 0.1 M isopropyl-*β*-d-thiogalactopyranoside was added to a final dilution of 0.4 mM, and the culture was incubated for another 2 h at 37 °C/220 rpm. After centrifugation for 10 min at 3200 × *g*, the supernatant was removed, and the pellet was resuspended in 10 ml of 0.1 M phosphate buffer (pH 7.2; 720 µl of 1 M Na_2_HPO_4_, 280 µl of 1 M NaH_2_PO_4_, 9 ml H_2_O). Cells were disrupted in a Potter–Elvehjem homogenisator, and by freeze thawing the suspension five times with liquid nitrogen. After centrifugation for 20 min at 3200 × *g*, the supernatant was collected, filtered through Miracloth (Merck, Germany) and stored at −80 °C for use in the RNAi experiment.

### RNA interference

Whole, expanding *G. rhellicani* inflorescences were cut at Ofenpass (Supplementary Table 1) on 17 July 2017 and transferred to the lab in water-filled vases. The dsRNA solution was mixed with carborundum (400 mesh, Sigma Aldrich, Switzerland) in a Petri dish, and 22 inflorescences were inoculated by rubbing one side of the inflorescences in the solution, leaving the other side of the inflorescence untreated. Four additional inflorescences were mock inoculated the same way using 0.1 M phosphate buffer instead of dsRNA solution. Inflorescences were kept at 21 °C/60% relative humidity/150 µmol m^−2^ S^−1^ light intensity at day (08:00 to 20:00) and 12 °C/60% relative humidity/dark at night. After 7 days, five flowers each from two dsRNA-treated plants showing a phenotype and the four control plants were collected in liquid nitrogen. Total RNA was extracted from these flowers, and 1 µg of RNA was treated with DNAse I as described above. DNAse I-treated RNA was converted to cDNA using the double volume of reagents as described above and split into two aliquots with and without the addition of reverse transcriptase. Reverse-transcription quantitative real-time PCR (RT-qPCR) was performed in triplicates on a CFX96 Touch™ Real-Time PCR Detection System (BioRad Laboratories, USA) using Maxima SYBR green (Thermo Fisher Scientific, USA). The PCR was set up as follows: 1.2 µl forward primer (5 µM, Supplementary Table 9), 1.2 µl reverse primer (5 µM, Supplementary Table 9), 0.5 µl ROX (0.25 µM), 10 µl Maxima SYBR Green Master Mix, 1 µl cDNA (congruent to 50 ng input RNA) and 6.1 µl ddH_2_O. The PCR conditions were: 95 °C for 10 min, 40 cycles of 95 °C for 15 s, 60 °C for 30 s, 72 °C/plate readout for 30 s. The three reference genes *glyceraldehyde 3-phosphate dehydrogenase* (*G3PDH*), *oligopeptidase* (*OPTD*) and *4-α-glucanotransferase* (*4-α-GTF*) have been used as control for RT-qPCR in related orchid species^79,80^. The reference genes were checked and showed low variation in expression between samples of our expression profiling dataset (see above). For each control and treatment plant, the quantification cycle (*C*_q_) was averaged over all three replicates per reference gene. The average *C*_q_ of all three reference genes was then subtracted from the average *C*_q_ of each target gene to obtain Δ*C*_q_. Ninety nine per cent confidence intervals were calculated for the Δ*C*_q_ values of the four control plants and compared to the Δ*C*_q_ values of each treatment plant.

### Code availability

All custom scripts are available from the corresponding authors upon request.

## Supplementary information

Supplementary Information

## Data Availability

The generated µCT data are deposited on Phaidra under accession number 912072, the UHPLC-MS/MS data are deposited on figshare under accession number 7321880, the mRNA-seq and Lexogen QuantSeq data are deposited on NCBI SRA under accession number PRJNA504609, the *GrMYB1* allele sequences are deposited in the NCBI Genbank nucleotide database under accession numbers MK163677–MK163681, and the *GrANS1* allele sequences under accession numbers MK163682–MK163685. All other datasets generated and analysed in this study are deposited on figshare under accession number 7314731. A reporting summary for this article is available as a Supplementary Information file.
