# Supplementary material for: Emergence of a floral colour polymorphism by pollinator-mediated overdominance
Source: Nat Commun. 2019 Jan 8;10:63. doi: 10.1038/s41467-018-07936-x (PMC6325131; doi:10.1038/s41467-018-07936-x)
Supplement: Supplementary file 1 — Supplementary Information [file 41467_2018_7936_MOESM1_ESM.pdf]

Supplementary Information

Emergence of a floral colour polymorphism by pollinator-mediated overdominance

Kellenberger *et al.*

Supplementary Figures

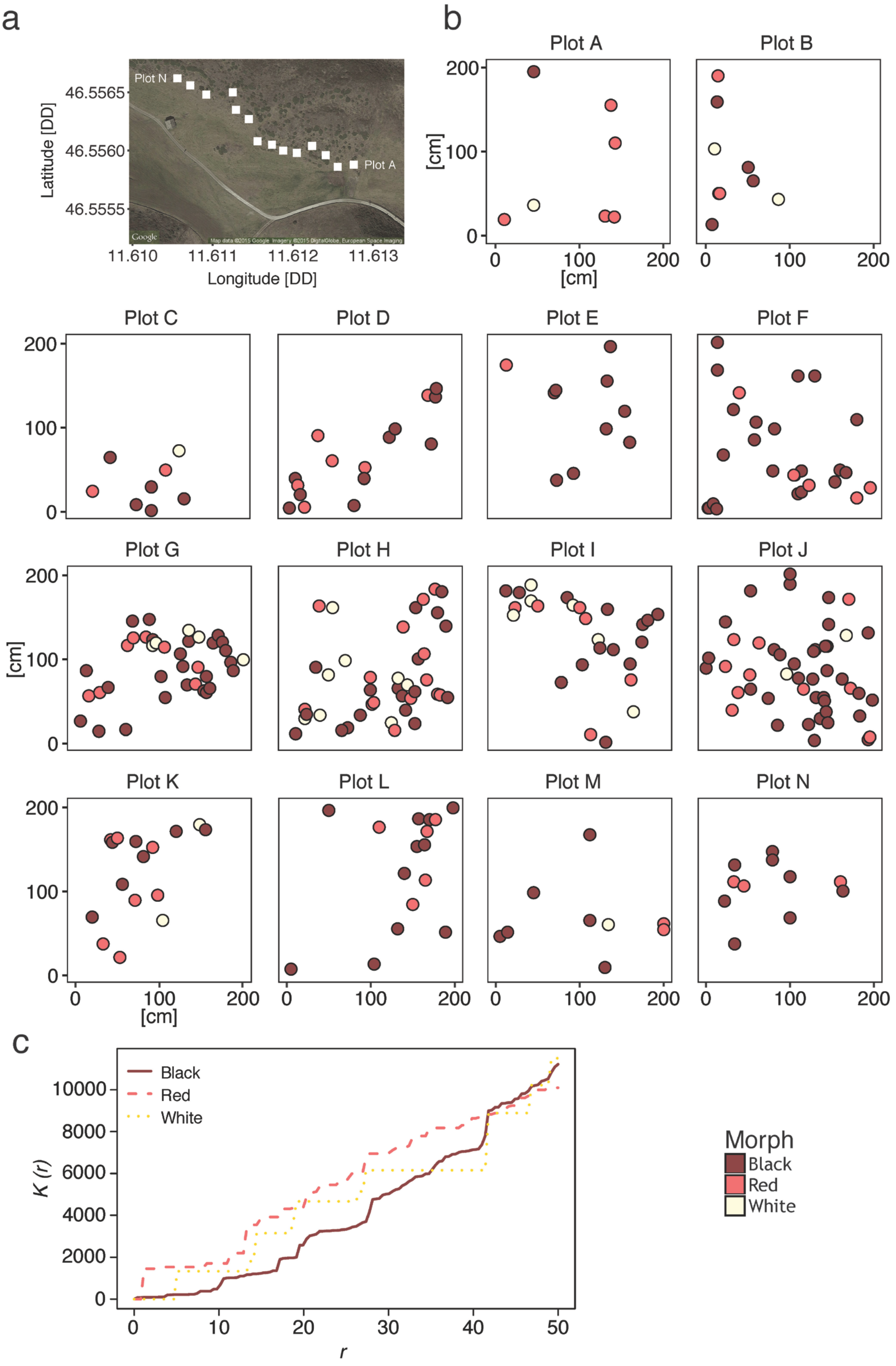

Supplementary Figure 1 | Spatial distribution of the *G. rhellicani* colour morphs within the experimental plots

a, Fourteen 2 × 2m plots (A-N) were established from east to west along the northern ridge of the *G. rhellicani* population on Puflatsch (satellite image from

Google Maps, 2018, Digital Globe, European Space Imaging; plot square sizes not to scale). **b**, Within the fourteen plots, the *G. rhellicani* plants are non-randomly distributed (Kolmogorov-Smirnov Test (n=281), $D = 0.19312$, $P = 1.578 \times 10^{-9}$): Overall plant density increases towards the centre of the population (plots G-J), and plants are outcompeted by other vegetation in certain plot areas. **c**, A Studentised Permutation Test shows that the spatial distribution pattern does not differ between the colour morphs (SPM-Test (n=281), $T = 127.09$, $P = 0.714$, lines show the mean of the summary function for each colour morph).

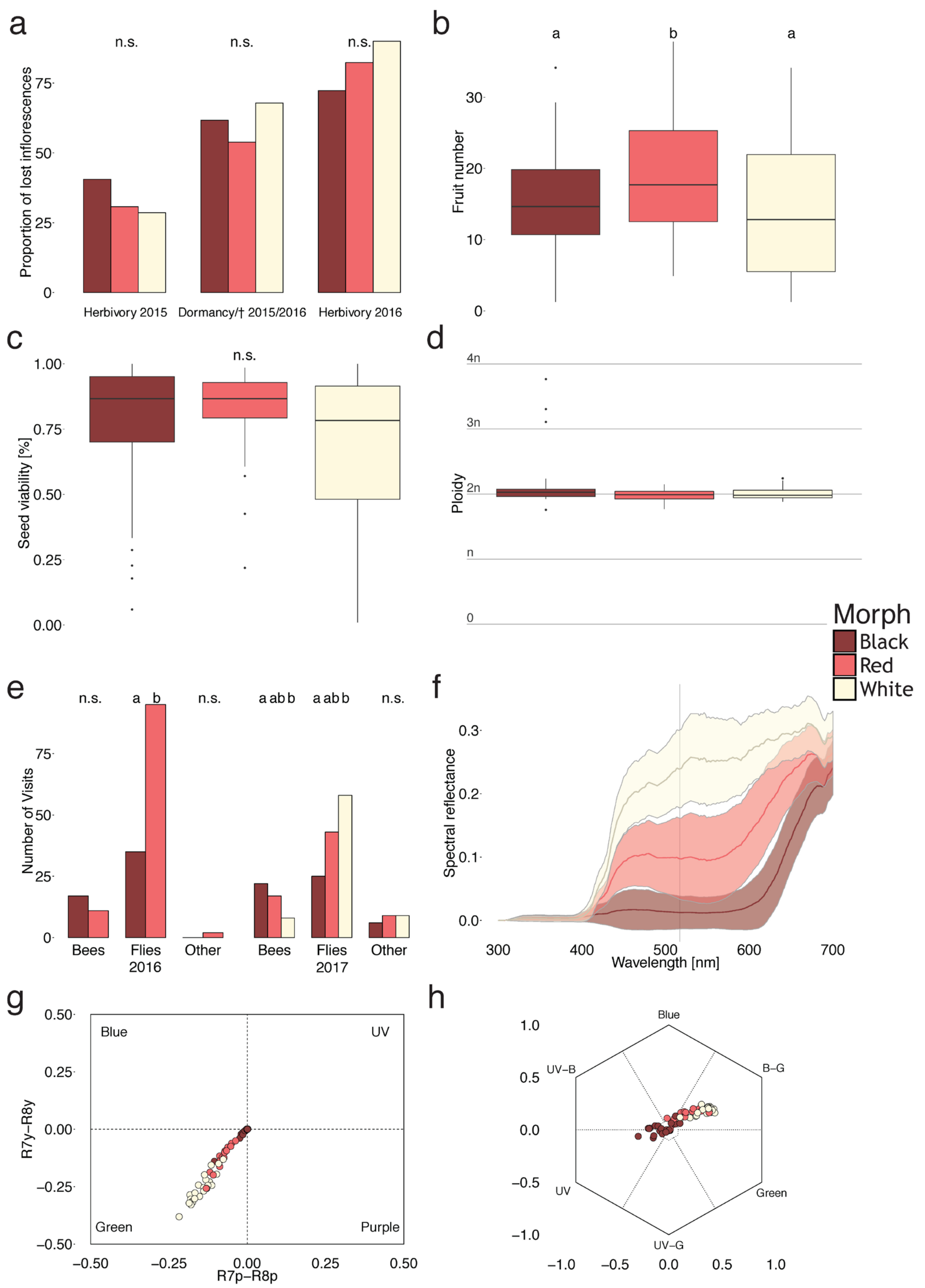

Supplementary Figure 2 | Ecological underpinnings of the colour polymorphism in *G. rhellicani*

a, Herbivory by insects and livestock does not differ between the colour morphs (higher in 2016 due to grazing by horses instead of cows, χ^2-test (n=281), χ^2=1.473, P=0.479), and all colour morphs are equally likely to disappear

(dormancy or death) in the next season (χ^2-test (n=281), χ^2=0.544, P=0.762). **b**, Red plants produce significantly more fruits than both black and white plants (ANOVA (n= 178), F(2, 175)=4.996, P=0.008, and TukeyHSD *post-hoc* test $t_{Black-Red}$=2.903, $P_{Black-Red}$=0.011, $t_{Black-White}$=-0.534, $P_{Black-White}$=0.851, $t_{Red-White}$=-2.359, $P_{Red-White}$=0.049); centre lines denote medians, bounds of boxes denote first and third quartiles, whiskers denote 1.5 × interquartile ranges. **c,** Seed viability does not differ between the colour morphs (Analysis of deviance of a generalized linear model with quasibinomial variance function (n=178), F(2, 175)=2.881, P=0.059); centre lines denote medians, bounds of boxes denote first and third quartiles, whiskers denote 1.5 × interquartile ranges. **d,** Apart from three seemingly polyploid individuals, flow cytometry identified all plants as diploid (shown for the Puflatsch population); centre lines denote medians, bounds of boxes denote first and third quartiles, whiskers denote 1.5 × interquartile ranges. **e**, Time lapse video recordings from 2016 and 2017 show that bees preferentially visit dark plants, while flies prefer bright plants (2016: χ^2-test bees (n=28), χ^2=1.286, P=0.257, χ^2-test flies (n=130), χ^2=27.692, P=1.422 × 10^{-7}, χ^2-test others (n=2), χ^2=2.000, P=0.157. 2017: χ^2-test bees (n=47), χ^2=6.426, P=0.040, χ^2-test flies (n=126), χ^2=13.000, P=0.002, χ^2-test others (n=24), χ^2=0.750, P=0.687). **f**, Mean ± 1 s.d. of the spectral reflectance of the three colour morphs from 300 to 700 nm. The vertical line indicates the absorbance maximum of the two main cyanidin pigments at 517 nm. **g**, Spectral reflectance of the colour morphs maps to the green quadrant of the fly visual space, with black plants positioned in the achromatic centre. **h**, Bees perceive *G. rhellicani* flowers mostly as blue-green, with the black and a subset of the red plants in the achromatic centre.

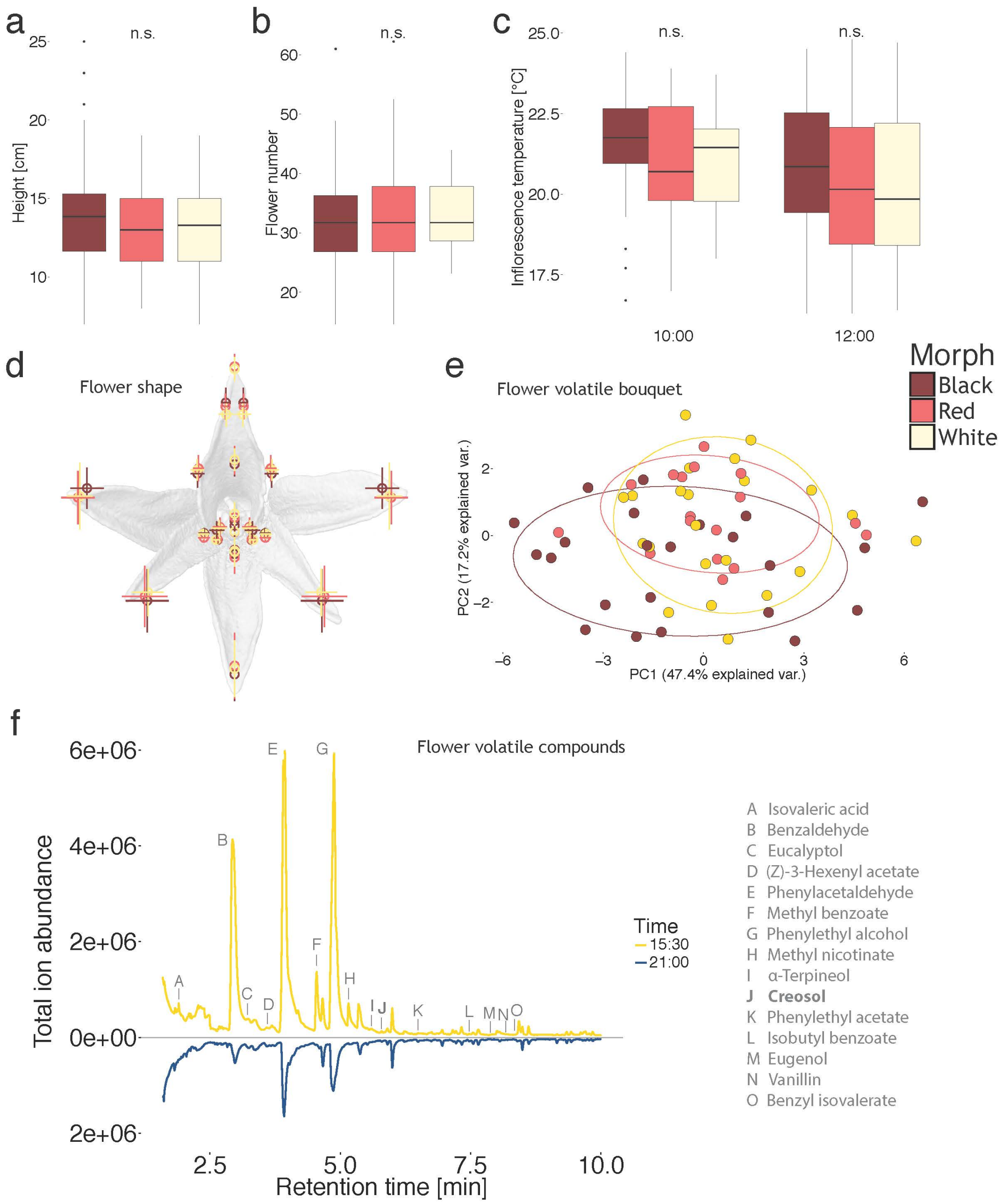

Supplementary Figure 3 | Differences in pollinator-relevant phenotypic traits between the *G. rhellicani* colour morphs

a, Inflorescence height does not differ between *G. rhellicani* colour morphs (ANOVA (n=343), *F*(2, 340)=1.341, *P*=0.263); centre lines denote medians, bounds of boxes denote first and third quartiles, whiskers denote 1.5 × interquartile ranges. **b**, Flower number per inflorescence/plant does not differ between colour morphs (ANOVA (n=271), *F*(2, 268)=0.342, *P*=0.710); centre lines denote medians, bounds of boxes denote first and third quartiles, whiskers denote 1.5 × interquartile ranges. **c**, Black plants do not heat up more than red or white morphs under solar radiation (10:00: ANOVA (n=60), *F*(2, 57)=0.328, *P*=0.722, 12:00: ANOVA (n=90), *F*(2, 87)=0.437, *P*=0.648); centre lines denote medians, bounds of boxes denote first and third quartiles, whiskers denote 1.5 ×

interquartile ranges. **d**, Flower shape is not significantly different between colour morphs (procrustes ANOVA of symmetrical component (n=48), $F(2, 45)=0.708$, $P=0.732$, procrustes ANOVA of asymmetrical component (n=48), $F(2, 45)=1.191$, $P=0.294$). The graphic shows the mean symmetrical procrustes coordinates ± 1 s.d. per morph. **e,** Principal component analysis showed no significant separation of colour morphs according to floral volatile bouquet. **f,** Floral volatile analysis identified 15 compounds contributing ≥ 0.1% to the floral bouquet, of which only the minor compound creosol showed a significantly reduced emission in red and white morphs (unlabelled peaks are contaminants also detected in the air control samples). After sunset, scent emission is strongly reduced in all colour morphs (blue curve). The well-known chocolate-like odour of *G. rhellicani* can be attributed to the presence of vanillin (peak N, see Supplementary Tables 2 and 5).

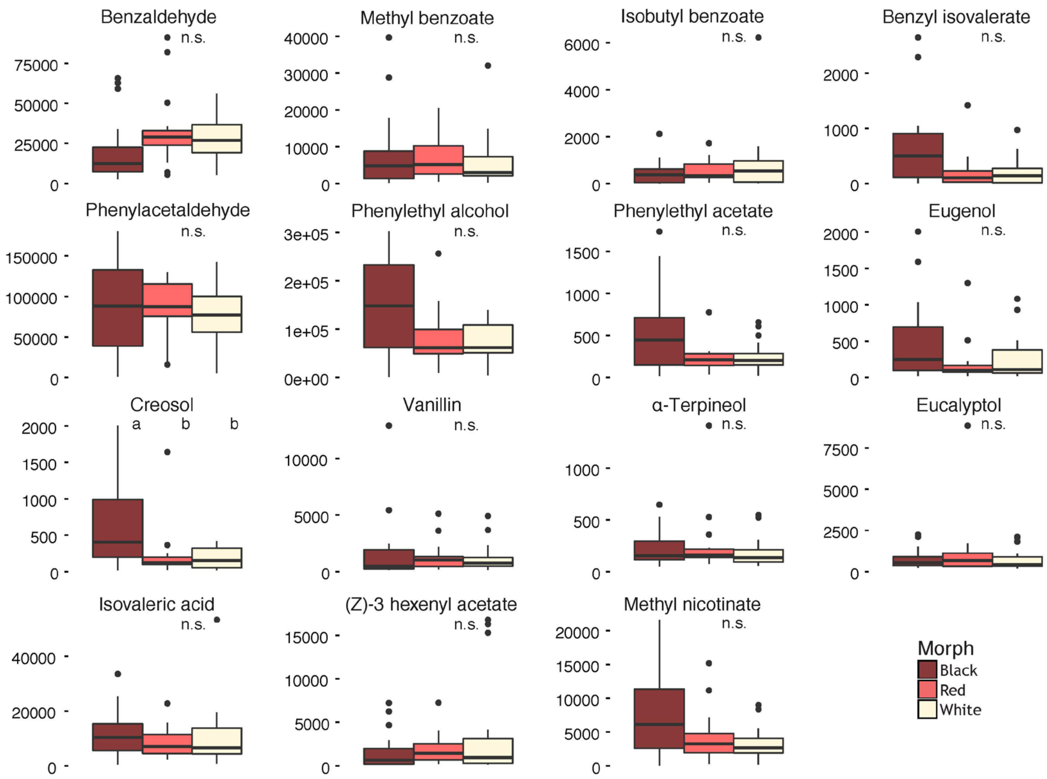

Supplementary Figure 4 | VOC quantities in the floral scent bouquet of the three *G. rhellicani* colour morphs

Emission rates in pg per l air of the 15 compounds which each account for ≥ 0.1% of the total floral scent bouquet recorded in the Puflatsch population. Apart from a trend towards a reduced emission of aromatic compounds in red and white morphs (only statistically significant for the minor compound creosol) there was no difference in VOC composition between the three colour morphs (see Supplementary Tables 2 and 5). Centre lines denote medians, bounds of boxes denote first and third quartiles, whiskers denote 1.5 × interquartile ranges.

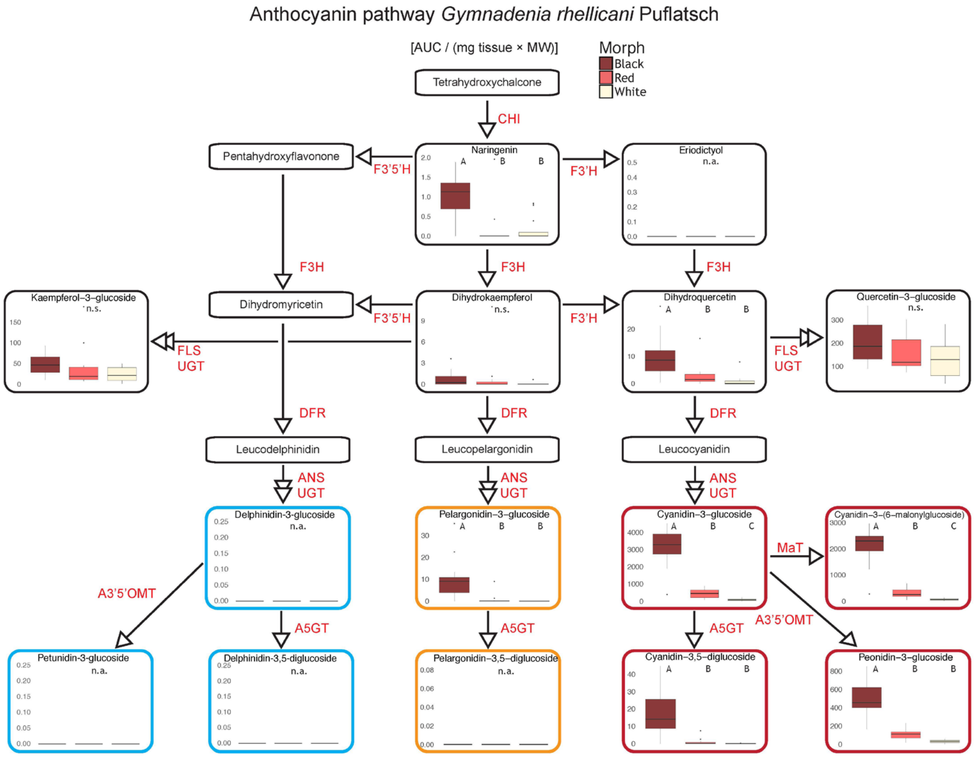

Supplementary Figure 5 | UHPLC-MS/MS quantifications of anthocyanins and precursors in flowers of the three *G. rhellicani* colour morphs

The anthocyanin pathway is divided into three branches producing blue delphinidin, orange pelargonidin, and red cyanidin pigments. Quantification of anthocyanins (area under curve (AUC) / (mg tissue × molecular weight (MW)), coloured frames) and colourless precursors (black frames) showed that the metabolite flux is mainly channelled through the cyanidin branch in *G. rhellicani*, hence the red colour of the plants. Further modifications such as the addition of different sugar residues and chemical groups can slightly change the hue of an anthocyanin compound. The red colour in *G. rhellicani* flowers is primarily due to cyanidin-3-glucoside, its derivative cyanidin-3-(6-malonylglucoside) and, to a lesser degree another derivative, peonidin-3-glucoside. The amount of the two main cyanidins is reduced more than 7.5 × in red, and more than 30 × in white colour morphs (ANOVA and TukeyHSD *post hoc* test, see Supplementary Table 6). All compounds were identified and quantified using authentic reference standards, except for cyanidin-3-(6-malonylglucoside), which was identified by an interpretation of the data spectrum obtained and based on previous reports (see Methods section in the main text). Standards for the two delphinidin precursors pentahydroxyflavonone and dihydromyricetin were unavailable, and leucodelphinidin, leucopelargonidin, and leucocyanidin are unstable intermediates. Enzyme abbreviations: CHI: chalcone isomerase, F3'5'H: flavonoid 3',5'-hydroxylase; F3'H: flavonoid 3'-hydroxylase; F3H: flavonoid 3-hydroxylase; FLS: flavonol synthase; UGT: UDP-glucose 3-*O*-glucosyltransferase; DFR: dihydroflavonol 4-reductase; ANS: anthocyanidin synthase; A3'5'OMT: A3',5'-*O*-

methyltransferase; A5GT: anthocyanin 5-*O*-glucosyltransferase; MaT: anthocyanin malonyl transferase. Centre lines denote medians, bounds of boxes denote first and third quartiles, whiskers denote 1.5 × interquartile ranges.

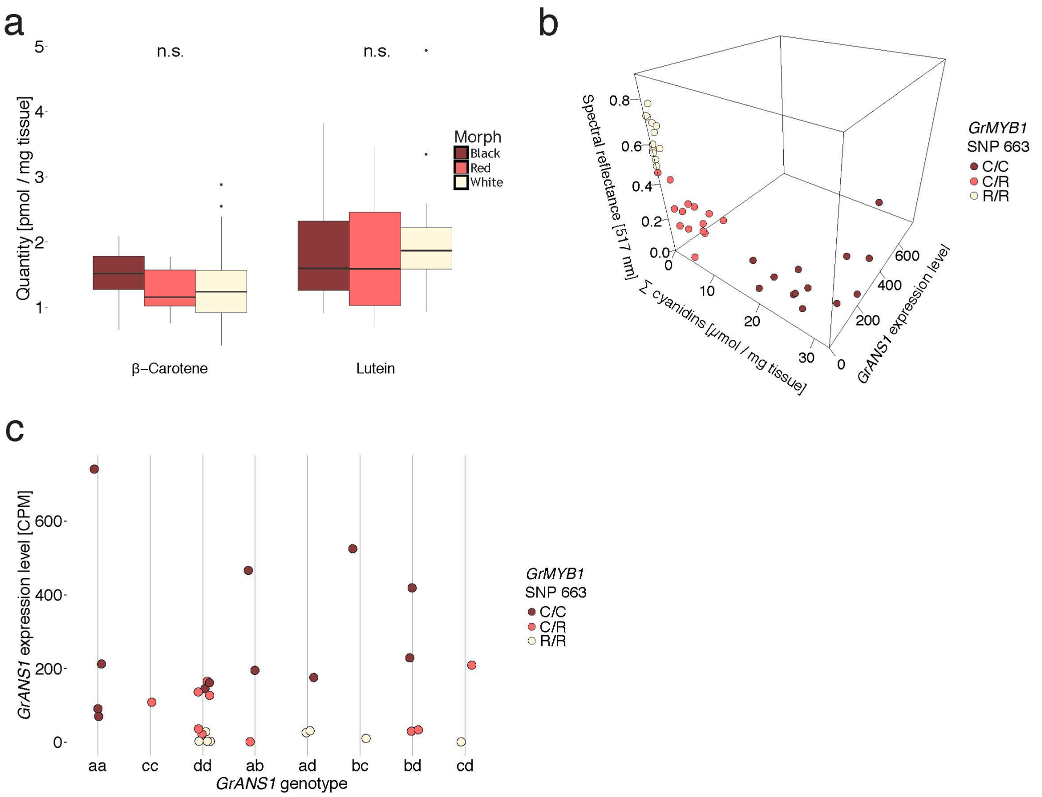

Supplementary Figure 6 | Quantification and correlation of floral pigments with the phenotype and genotype of the three *G. rhellicani* colour morphs

a, Concentration of the two main floral carotenoids, β-carotene and lutein, is not different between the colour morphs (β-carotene: ANOVA (n=43), $F(2, 40)=0.520$, $P=0.598$, lutein: ANOVA (n=45), $F(2, 42)=0.614$, $P=0.546$); centre lines denote medians, bounds of boxes denote first and third quartiles, whiskers denote 1.5 × interquartile ranges. **b**, Correlation between phenotype (spectral reflectance at 517 nm), metabolomics (combined amount of the two main cyanidins), and transcriptomics data (*GrANS1* expression level). Samples are coloured according to genotype (SNP at position 663 in the *GrMYB1* transcript, R = A or G). **c,** *GrANS1* genotype is neither correlated with *GrANS1* expression level, nor with plant colour (*GrMYB1* genotype). Letters a-d denote different *GrANS1* alleles identified by a combination of nine SNPs in the coding sequence (SNP states of allele a: GGGGCACTC, allele b: GGGGTACCC, allele c: GAAGCGATG, allele d: AGGTCGACG).

Supplementary Tables

Supplementary Table 1 | Coordinates of the populations assessed in this study

Table showing the country, latitude and longitude coordinates in WGS 84 format, as well as the elevation in metres above sea level (m.a.s.l.) of the assessed *G. rhellicani* populations (sorted from west to east).

Population	Country	Latitude	Longitude	Elevation [m.a.s.l.]
Chandolin	Switzerland	46° 15' 11.3'' N	07° 36' 39.2'' E	2290
Furkapass	Switzerland	46° 35' 14.0'' N	08° 26' 10.2'' E	2255
Albulapass	Switzerland	46° 34' 53.2'' N	09° 48' 50.2'' E	2210
Davos	Switzerland	46° 48' 39.9'' N	09° 49' 20.9'' E	1980
Berninapass	Switzerland	46° 26' 43.5'' N	09° 58' 33.4'' E	2080
Ofenpass	Switzerland	46° 38' 02.4'' N	10° 17' 00.5'' E	2295
Reschenpass	Italy	46° 49' 13.9'' N	10° 32' 44.9'' E	2270
Rifflsee	Austria	46° 58' 03.9'' N	10° 50' 10.3'' E	2255
Bondone	Italy	46° 00' 25.8'' N	11° 01' 39.7'' E	1695
Puflatsch	Italy	46° 33' 22.4'' N	11° 36' 37.6'' E	2110
Sajatkopf	Austria	47° 01' 57.0'' N	12° 22' 20.0'' E	2320
Edelweißspitze	Austria	47° 07' 23.0'' N	12° 49' 30.0'' E	2305
Astental	Austria	46° 57' 35.7'' N	12° 57' 26.4'' E	2055
Heiligenbachalm	Austria	46° 56' 34.3'' N	13° 44' 35.2'' E	1925
Stubalpe	Austria	47° 05' 08.0'' N	14° 56' 05.0'' E	1635

Supplementary Table 2 | Sample sizes of the experiments conducted in this study

Table showing the number of black, red, and white *G. rhellicani* plants per population (Puflatsch, Bondone, and all other populations in total), which were used in the experiments of this study. Analyses marked with an asterisk (*) included the same 48 RFID-marked focal plants from Puflatsch (or a subset thereof).

Experiment	Puflatsch			Bondone			Other	Total
	Black	Red	White	Black	Red	White	Black	
Colour persistence	67	36	9	0	0	0	0	112
Plant survival	175	78	28	0	0	0	263	544
Reproductive fitness	101	52	20	0	0	0	0	173
Flow cytometry*	23	14	20	4	13	0	179	253
Pollinator exclusion	0	0	0	0	0	0	40	40
Pollinator recordings 2016	11	11	0	0	0	0	0	22
Pollinator recordings 2017	10	10	10	0	0	0	0	30
Spectral mapping*	23	15	26	7	24	1	153	249
Height*	198	91	54	7	24	1	406	781
Temperature	50	50	50	0	0	0	0	150
3D morphometrics*	16	16	16	0	0	0	101	149
Floral volatile analysis*	23	15	24	7	24	1	155	249
Floral volatile time course	7	0	7	0	0	0	7	21
UHPLC anthocyanins*	16	14	17	7	24	1	189	268
HPLC carotenoids*	15	14	16	7	24	1	30	107
Transcriptome assembly*	1	1	1	0	0	0	3	6
mRNA expression profiles*	14	14	15	0	0	0	33	76
Differential expression*	14	14	14	0	0	0	0	42
TWAS*	14	14	15	0	0	0	0	43
GrMYB1 Genotyping*	31	26	31	7	15	1	75	186
GrANS1 Genotyping*	12	10	8	0	0	0	0	30
RNAi	0	0	0	0	0	0	26	26

Supplementary Table 3 | Insect taxa recorded on *G. rhellicani* inflorescences at Puflatsch

Insects were identified (K.J.R.P.B.) down to family, and if possible, to genus or species level. Numbers in brackets indicate insects with pollinia attached. The 2015 dataset was compiled by catching insects observed on *G. rhellicani* inflorescences along east-west transects on both ridges in the morning (10:00) and after sunset (22:00). Numbers of these recordings may thus be biased by insect visibility and netting skill. Also, all listed observations are from 10:00 as no insect landings were recorded after sunset. The 2016 and 2017 datasets are based on the time-lapse camera recordings and should thus be unbiased. 2016 camera data was only collected from black and red plants.

**Collection 2015**					
**Order**	**Family or higher taxonomic group**	**Species**	**# on Black**	**# on Red**	**# on White**
Coleoptera	Cryptophagidae	*Atomaria* sp.	0	1 (0)	0
Coleoptera	Rutelidae	*Phyllopertha horticola*	1 (0)	0	0
Diptera	Anthomyiidae	Unknown	3 (0)	2 (0)	0
Diptera	Empididae	Unknown	1 (0)	0	1 (0)
Diptera	Muscidae	Unknown	11 (2)	6 (1)	4 (0)
Diptera	Rhagonidae	*Symphoromyia crassicornis*	1 (0)	0	0
Hymenoptera	Apidae	*Apis mellifera*	0	0	1 (0)
Hymenoptera	Apidae	*Bombus monticola*	2 (2)	1 (0)	1 (0)
Hymenoptera	Apidae	*Bombus rupestris*	1 (0)	0	0
Lepidoptera	Crambidae	*Catoptria* sp.	1 (0)	0	0
Lepidoptera	Crambidae	*Crambus pratellus*	8 (2)	1 (0)	1 (0)
Lepidoptera	Crambidae	*Crambus* sp.	3 (1)	0	1 (0)
Lepidoptera	Crambidae	Unknown	1 (0)	0	0
Lepidoptera	Gelechoidea (superfamily)	Unknown	1 (0)	3 (2)	2 (1)
Lepidoptera	Noctuidae	Unknown	1 (1)	0	0
Lepidoptera	Nymphalidae	*Coenonympha gardetta*	0	1 (1)	1 (0)
Lepidoptera	Nymphalidae	*Coenonympha pamphilus*	0	0	1 (1)
Lepidoptera	Nymphalidae	*Erebia mnestra*	1 (0)	0	0
Lepidoptera	Zygaenidae	*Zygaena exulans*	3 (1)	0	0

Time-lapse cameras 2016					
**Order**	**Family or higher taxonomic group**	**Species**	**# on Black**	**# on Red**	**# on White**
Coleoptera	Unknown	Unknown	0	2	-
Diptera	Empididae or Bibionidae	Unknown	0	1	-
Diptera	Muscidae	Unknown	2	11	-
Diptera	Rhagonidae	Unknown	0	7	-
Diptera	Syrphidae	*Eristalis* sp	1	4	-
Diptera	Syrphidae	*Sphaerophoria* sp.	1	3	-
Diptera	Syrphidae	Unknown Syrphini	2	7	-
Diptera	Syrphidae	Unknown	2	2	-
Diptera	Unknown	Unknown	27	60	-
Hymenoptera	Apidae	*Apis mellifera*	10	11	-
Hymenoptera	Apidae	*Bombus* sp.	6	0	-
Hymenoptera	Anthophila (clade)	Unknown	1	0	-

Time-lapse cameras 2017					
**Order**	**Family or higher taxonomic group**	**Species**	**# on Black**	**# on Red**	**# on White**
Coleoptera	Unknown	Unknown	1	4	5
Diptera	Muscoidea (superfamily)	Unknown	7	5	5
Diptera	Sarcophagidae	Unknown	1	0	1
Diptera	Syrphidae	*Eristalis tenax*	2	0	2
Diptera	Syrphidae	Unknown Syrphini	0	2	6
Diptera	Syrphidae	Unknown	1	3	1
Diptera	Unknown	Unknown	17	34	53
Hymenoptera	Apidae	*Apis mellifera*	21	14	7
Hymenoptera	Apidae	*Bombus* sp.	1	1	1
Hymenoptera	Anthophila (clade)	Unknown	0	2	0
Lepidoptera	Hesperiidae	*Pyrgus* sp.	0	0	1
Lepidoptera	Nymphalidae	*Coenonympha gardetta*	0	1	0
Lepidoptera	Nymphalidae	*Coenonympha pamphilus*	0	1	0
Lepidoptera	Nymphalidae	*Coenonympha* sp.	0	1	1
Lepidoptera	Microlepidoptera	Unknown	3	2	2

Supplementary Table 4 | ANOVA tables from the linear mixed-effects models of floral VOC emission in *G. rhellicani* colour morphs

Prior to analysis, all VOC data was BoxCox transformed to approach normality. Differences in VOC emission between the colour morphs were modelled using Holm-adjusted linear mixed-effects models with sampling year as random factor (n=62), and Tukey HSD *post-hoc* tests. % bouquet: average proportion of the entire floral volatile bouquet, F (2,58): conditional F-statistic, P_{adj}: Holm-adjusted *P*-value (P<0.05 in bold), *post-hoc*: summary of TukeyHSD *post-hoc* comparisons (B: black, R: red, W: white, n.a. = *post-hoc* test not applicable).

**Compound**	**% bouquet**	**F (2,58)**	P_{adj}	***post-hoc***
Isovaleric acid	5.1	0.342	1.000	n.a.
Benzaldehyde	13.2	4.507	0.212	n.a.
Eucalyptol	0.6	0.500	1.000	n.a.
(*Z*)-3 hexenyl acetate	1.2	0.574	1.000	n.a.
Phenylacetaldehyde	35.1	0.159	1.000	n.a.
Methyl benzoate	2.9	0.128	1.000	n.a.
Phenylethyl alcohol	38.3	4.055	0.292	n.a.
Methyl nicotinate	1.9	3.032	0.615	n.a.
α-Terpineol	0.1	0.552	1.000	n.a.
Creosol	0.2	8.126	**0.012**	t_{B-R} = -3.122, P_{B-R} = **0.005**
				t_{B-W} = -3.693, P_{B-W} **< 0.001**
				t_{R-W} = -0.318, P_{R-W} = 0.946
Phenylethyl acetate	0.1	2.456	0.946	n.a.
Isobutyl benzoate	0.5	1.956	1.000	n.a.
Eugenol	0.1	2.439	0.946	n.a.
Vanillin	0.6	0.117	1.000	n.a.
Benzyl isovalerate	0.1	3.223	0.561	n.a.

Supplementary Table 5 | Flavonoids identified in *G. rhellicani* flowers

Table showing the physical and chemical properties of all flavonoids identified in this study. Naringenin, dihydrokaempferol and pelargonidin-3-glucoside were present at low intensity, whereas eriodictyol, pelargonidin-3,5-diglucoside, and delphinidin compounds were not detected. EC: elemental composition, RT: retention time (min), m/z: observed mass by charge, Mass: theoretical mass [M]+/[M+H]+, MSMS: tandem mass spectrometry fragments (n.f.: no fragmentation under the present experimental conditions), λ_{max}: absorbance peaks in the UV/Vis spectrum in nm (NA: no λ_{max} detected due to low intensity).

Compound	EC	RT	m/z	Mass	MSMS	λ_{max}
Naringenin	$C_{15}H_{12}O_5$	7.90	273.0761	273.0758	**273**→ 153, 147, 119	NA
Kaempferol 3-glucoside	$C_{21}H_{20}O_{11}$	6.22	449.1080	449.1078	**449**→ 287	264, 346
Dihydrokaempferol	$C_{15}H_{10}O_6$	8.16	287.0554	287.0550	n.f.	NA
Quercetin 3-glucoside	$C_{21}H_{20}O_{12}$	5.96	465.1036	465.1028	**465**→ 303	256, 352
Dihydroquercetin	$C_{15}H_{10}O_7$	7.42	303.0499	303.0499	n.f.	254, 370
Pelargonidin 3-glucoside	$C_{21}H_{21}O_{10}$	4.97	433.1140	433.1129	**433**→ 271	NA
Cyanidin 3-glucoside	$C_{21}H_{21}O_{11}$	4.73	449.1073	449.1078	**449**→ 287	208,280,516
Cyanidin 3,5-diglucoside	$C_{27}H_{31}O_{16}$	4.18	611.1609	611.1607	**611**→ 449, 287	276, 518
Peonidin 3-glucoside	$C_{22}H_{23}O_{11}$	5.01	463.1200	463.1235	**463**→ 301	276, 516
Cyanidin 3-(6-malonylglucoside)	$C_{24}H_{23}O_{14}$	5.10	535.1087	535.1082	**535**→ 287	280,516

Supplementary Table 6 | ANOVA table of flavonoid quantities in *G. rhellicani* colour morphs

Relative quantities of anthocyanins and precursor compounds were calculated from the UHPLC-MSMS chromatograms as area under curve (AUC) / mg tissue × molecular weight (MW), and BoxCox transformed to approach normality. Differences in anthocyanin metabolite quantities between the colour morphs were modelled using Holm-adjusted ANOVAs (n=47), and Tukey HSD *post-hoc* tests. Delphinidin derivatives, eriodictyol, and pelargonidin-3,5-diglucoside were not detected in the samples and were thus excluded from the analysis. F (2,44): conditional F-statistic, P_{adj}: Holm-adjusted P-value (P<0.05 in bold), *post-hoc*: summary of TukeyHSD *post-hoc* comparisons (B: black, R: red, W: white, n.a. = ANOVA / *post-hoc* test not applicable).

Compound	F (2,44)	P_{adj}	*post-hoc*
Naringenin	14.118	**9.17×10^{-5}**	t_{B-R} = -4.603, P_{B-R} **< 0.001**<br>t_{B-W} = -4.575, P_{B-W} **< 0.001**<br>t_{R-W} = 0.102, P_{R-W} = 0.994
Eriodictyol	n.a.	n.a.	n.a.
Kaempferol-3-glucoside	4.016	0.075	n.a.
Dihydrokaempferol	1.117	0.336	n.a.
Quercetin-3-glucoside	3.689	0.075	n.a.
Dihydroquercetin	12.213	**2.42×10^{-4}**	t_{B-R} = -3.610, P_{B-R} **= 0.002**<br>t_{B-W} = -4.715, P_{B-W} **< 0.001**<br>t_{R-W} = -1.028, P_{R-W} = 0.564
Delphinidin-3-glucoside	n.a.	n.a.	n.a.
Delphinidin-3,5-diglucoside	n.a.	n.a.	n.a.
Petunidin-3-glucoside	n.a.	n.a.	n.a.
Pelargonidin-3-glucoside	12.213	**1.75×10^{-5}**	t_{B-R} = -4.808, P_{B-R} **< 0.001**<br>t_{B-W} = -5.306, P_{B-W} **< 0.001**<br>t_{R-W} = -0.412, P_{R-W} = 0.911
Pelargonidin-3,5-diglucoside	n.a.	n.a.	n.a.
Cyanidin-3-glucoside	97.900	**5.86×10^{-16}**	t_{B-R} = -7.387, P_{B-R} **< 0.001**<br>t_{B-W} = -13.981, P_{B-W} **< 0.001**<br>t_{R-W} = -6.366, P_{R-W} **< 0.001**
Cyanidin-3,5-diglucoside	28.687	**7.42×10^{-8}**	t_{B-R} = -6.313, P_{B-R} **< 0.001**<br>t_{B-W} = -6.750, P_{B-W} **< 0.001**<br>t_{R-W} = -0.328, P_{R-W} = 0.943
Peonidin-3-glucoside	76.207	**4.07×10^{-14}**	t_{B-R} = -9.473, P_{B-R} **< 0.001**<br>t_{B-W} = -11.552, P_{B-W} **< 0.001**<br>t_{R-W} = -1.891, P_{R-W} = 0.153
Cyanidin-3-(6-malonylglucoside)	98.299	**5.86×10^{-16}**	t_{B-R} = -7.422, P_{B-R} **< 0.001**<br>t_{B-W} = -14.008, P_{B-W} **< 0.001**<br>t_{R-W} = -6.358, P_{R-W} **< 0.001**

Supplementary Table 7 | Transcripts differentially expressed between black and white *G. rhellicani* colour morphs

Two of the thirteen transcripts differentially expressed between black and white *G. rhellicani* colour morphs (rank 1 and 10) map to the same *anthocyanidin synthase* (*ANS*) transcript. Analysis with *edgeR* v. 3.12.1 (n=42); logFC: absolute log_2-fold changes between average expression levels per morph, usr-logFC: Un-shrunk log_2-fold expression level changes between morphs, logCPM: average log_2 counts per million, *P*-adj: *P*-value adjusted with False Discovery Rate (FDR) correction ($P<0.05$ in bold).

Transcript	BLASTn best hit	logFC	usr-logFC	logCPM	*P*-adj
TR261401.c0_g1	*anthocyanidin synthase* (*ANS*)	4.155	4.168	6.925	**1.72×10^{-4}**
TR112617.c0_g3	no hit	3.894	3.952	4.486	**1.90×10^{-4}**
TR224389.c1_g2	no hit	4.343	4.353	7.309	**0.004**
TR262779.c6_g4	no hit	3.378	3.425	4.549	**0.016**
TR234590.c1_g6	no hit	3.769	4.839	1.905	**0.016**
TR200635.c1_g9	no hit	4.911	5.158	3.744	**0.016**
TR202234.c2_g5	no hit	3.280	3.320	4.468	**0.025**
TR252418.c4_g3	*sulphate transporter*	2.794	2.941	2.690	**0.027**
TR218853.c1_g6	*peptidyl-prolyl cis-trans isomerase*	3.623	3.868	2.851	**0.027**
TR126623.c0_g1	*anthocyanidin synthase* (*ANS*)	5.860	5.881	7.658	**0.033**
TR261727.c1_g5	*no hit*	-3.222	-3.433	2.807	**0.045**
TR250925.c2_g5	*no hit*	-4.757	-7.305	2.032	**0.045**
TR262554.c4_g7	*no hit*	-5.550	-1.443	2.446	**0.045**

Supplementary Table 8 | Top ten SNPs associated with floral spectral reflectance at λ = 517 nm of the *G. rhellicani* colour morphs

Three of the top ten SNPs associated with spectral reflectance at 517 nm wavelength, the absorption maximum of cyanidins, are situated in the 3'-UTR of the same *R2R3-MYB* transcription factor. Analysis with GAPIT v. 2 (n=43); MAF: Frequency of the minor allele, R^2-exc: R^2 of the model excluding the SNP, R^2-inc: R^2 of the model including the SNP, *P*: (unadjusted) *P*-value.

Transcript	Position	BLASTn best hit	MAF	R^2-exc	R^2-inc	*P*
TR259745.c4_g1	177	no hit	0.233	0.397	0.694	**2.08×10^{-4}**
TR224083.c24_g2	59	uncharacterised	0.477	0.397	0.672	**3.28×10^{-4}**
TR272909.c1_g9	777	uncharacterised	0.477	0.397	0.612	**1.21×10^{-3}**
TR101194.c0_g1	1623	no hit	0.430	0.397	0.604	**1.44×10^{-3}**
TR255984.c3_g4	375	*snRNP*	0.453	0.397	0.603	**1.46×10^{-3}**
TR255984.c3_g4	376	*snRNP*	0.453	0.397	0.603	**1.46×10^{-3}**
TR237780.c2_g1	968	*R2R3-MYB*	0.465	0.397	0.601	**1.54×10^{-3}**
TR237780.c2_g1	978	*R2R3-MYB*	0.453	0.397	0.591	**1.96×10^{-3}**
TR237780.c2_g1	886	*R2R3-MYB*	0.465	0.397	0.590	**1.98×10^{-3}**
TR263130.c2_g1	155	no hit	0.256	0.397	0.589	**2.03×10^{-3}**

Supplementary Table 9 | Primer sequences used in this study

Table showing primer sequences used for genotyping, cloning, and RT-qPCR. For qRT-PCR primers, an additional melting-curve analysis was performed to assess binding specificity (F = forward primer, R = reverse primer).

**Primer sequences used for genotyping (5' → 3')**	
GrMYB1-F	ATGGAGAGAGAGAGGGGGG
GrMYB1-R	TCATTGTTCCCAATCAACATC
GrMYB1-F2 (last exon)	TAATGCATGGACAGCAACAG
GrANS1-F	ATGGCAGCCAAAATCACTCCC
GrANS1-R	TCAATTCGCAGCGGCTGCC
GrANS1-Fi (internal)	CCGACTACATTGAGGCAACGAC
GrANS1-Ri (internal)	TCGTTGCCTCAATGTAGTCGG
**Primer sequences used for cloning (attB-sites in capital, 5' → 3')**	
GrMYB1-attB-F	GGGGACAAGTTTGTACAAAAAAGCAGGCTAAtaatgcatggacagcaacag
GrMYB1-attB-R	GGGGACCACTTTGTACAAGAAAGCTGGGTCCTAtcattgttcccaatcaacatcaag
**Primer sequences used for qRT-PCR (5' → 3')**	
GrMYB1-qPCR-F	CGACCCGACGATATTAATGC
GrMYB1-qPCR-R	CGGATCAAAGGATTCAAAGC
GrANS1-qPCR-F	GCGAATTGAACGGGAGAAG
GrANS1-qPCR-R	AAGATTGCCCAAGTTCAAGC
GrG3PDH-qPCR-F	TCCTCAGGATTTCTAACCCCAAAG
GrG3PDH-qPCR-R	CTTCATCACCACCGAATACATGAC
GrOPTD-qPCR-F	GAGCTGCACTTTCTACATCTGATG
GrOPTD-qPCR-R	CACTTGTAGCATCCCCAAAGAAAC
Gr4αGTF-qPCR-F	CTTCTTGTGTCCATCCTGTTATGC
Gr4αGTF-qPCR-R	GCTCACTTGGCATTCTCTGAATAC
